# Engineering a switchable single‐chain TEV protease to control protein maturation in living neurons

**DOI:** 10.1002/btm2.10292

**Published:** 2022-02-22

**Authors:** Pietro Renna, Cristian Ripoli, Onur Dagliyan, Francesco Pastore, Marco Rinaudo, Agnese Re, Fabiola Paciello, Claudio Grassi

**Affiliations:** ^1^ Department of Neuroscience Università Cattolica del Sacro Cuore Rome Italy; ^2^ Fondazione Policlinico Universitario A. Gemelli IRCCS Rome Italy; ^3^ Department of Neurobiology Harvard Medical School Boston Massachusetts USA

**Keywords:** dendritic spines, neuronal plasticity, neurotrophins, protease, protein engineering, TEV

## Abstract

Engineered proteases are promising tools to address physiological and pathophysiological questions as well as to develop new therapeutic approaches. Here we introduce a new genetically encoded engineered single‐chain tobacco etch virus protease, allowing to control proprotein cleavage in different compartments of living mammalian cells. We demonstrated a set of controllable proteolytic effects, including cytosolic protein cleavage, inducible gene expression, and maturation of brain‐derived neurotrophic factor (BDNF) in the secretory pathway thus showing the versatility of this technique. Of note, the secretory pathway exhibits different characteristics from the cytosol and it is difficult to target because inaccessible to some small molecules. We were able to induce ligand‐mediated BDNF maturation and monitor its effects on dendritic spines in hippocampal pyramidal cells and in the mouse brain. This strategy paves the way to dissect proteolytic cleavage product signaling in various processes as well as for future therapeutic applications.

## 1 INTRODUCTION

Most growth factors, hormones, and functional proteins are synthesized as proproteins and cleaved by specific proteases in response to physiological stimuli. The human genome encodes hundreds of proteases,^1^ producing thousands of proteolytic cleavage products, but the role of most of them is unclear.^2^

In the central nervous system, proteolytic cleavage products either affect the cells they are released from (autocrine signaling) or diffuse to modulate the function of other cells (paracrine signaling). Such modulatory effects have been suggested to play a central role in the function of neuronal circuits.^2^ One way to study the roles of proteolytic cleavage products is to apply the purified protein to the system. This approach is effective in vitro but it is not useful to dissect the autocrine and paracrine modulations, as well as the effects of pre‐ versus postsynaptic mature product secretion on neuronal function. Moreover, it has been a challenge to deliver the purified proteins in vivo, especially in a cell type‐specific manner.^3^, ^4^ Another challenge is that multiple proteolytic cleavage products can be made from a single precursor gene, thus genetic knockout (KO) and knockdown (KD) can fall short for cleavage product‐specific perturbations. Also, molecular compensations due to the slow actions of KO and KD over days and the lack of broadly applicable genetically encoded acute perturbation techniques necessitate new approaches.

A critical step in the active factor production and sorting is the spatiotemporal activity of the specific proteases, which cleave precursor proproteins to form the mature products. Therefore, we focused on this step to develop a tool controlling the maturation process. One of the most widely used proteases in biotechnology is the stable and mammalian‐safe NIa tobacco etch virus (TEV) protease.^5^ Endogenous proteases cleave protein targets at an optimum range of pH in different compartments of the cells.^6^ Thus, the highly specific activity of TEV at broad pH and temperature ranges renders this protease an ideal candidate to cleave proteins at different conditions, offering new therapeutic and biotechnological applications. Moreover, the recent overcoming of the major TEV limit, that is, its slow catalytic activity, further boosts the potential application of this protease for regulating biological processes.^7^ Although TEV has been previously engineered for chemogenetic control of proteolytic activity, the approaches used so far are limited to protein‐fragment complementation.^8^, ^9^, ^10^ Of note, the major drawbacks of two‐component split systems are that they are subjected to variable stoichiometry, exhibit high background activity and heterogeneous proteolytic response.^11^ These limitations have hampered so far the application of engineered TEV to control the maturation, activation, and inactivation of proteins in vivo.

Here, we introduce a new genetically encoded switchable single‐chain TEV allowing a chemically inducible cleavage of proteins containing the TEV cleavage site (TEVcs) in different compartments of cells. We demonstrate that the genetically encoded switchable single‐chain TEV can be robustly activated in the secretory pathway of cells to cleave proproteins. As a proof‐of‐concept, we use this new tool to cut cytosolic and membrane‐localized synthetic proteins controlling gene expression and proBDNF in the secretory pathway producing brain‐derived neurotrophic factor (BDNF) that increased dendritic spine volume and density in hippocampal CA1 pyramidal neurons and extracellular signal‐regulated kinase (ERK) phosphorylation after its injection into mouse hippocampi.

## 2 MATERIALS AND METHODS

### 2.1 Ethics and animal use statement

All animal procedures were approved by the Ethics Committee of Università Cattolica del Sacro Cuore and were fully compliant with Italian (Ministry of Health guidelines, Legislative Decree No. 116/1992) and European Union (Directive No. 86/609/EEC) legislation on animal research. The methods were carried out in strict accordance with the approved guidelines.

### 2.2 DNA plasimds and molecular cloning

To avoid autoproteolysis, TEV and secTEV constructs used in this work contain S219V^12^ mutation and NEGGLE peptide,^13^ respectively. Sequence mutations (MRVRRH to ENLYFQ in proBDNF) were done with QuikChange II system (Agilent Technologies). Insertions of uniRapR sequences into TEV and secTEV were obtained through the Gibson Assembly Reaction (Gibson Cloning MasterMix, NEB). All restriction enzymes were purchased from New England Biolabs. The crystal structure of TEV was obtained from the Protein data bank (PDB ID: 1LVM). Plasmidic DNA was directly sequenced on both strands using BigDyeTerminator V3.1 (Applied Biosystems) and subsequently resolved on SAM solution (Applied Biosystems). Sequences data were inspected using Sanger sequencing and Fragment Analysis Software SeqScape of Applied Biosystems (Thermo Fisher Scientific) and assisted by SnapGene software (GSL Biotech). Primers amplifying uniRapR fragment and TEV plasmids are shown in Table S3.

### 2.3 HEK293T cell culture

HEK293T cells were cultured in high glucose Dulbecco's modified Eagle medium (Sigma‐Aldrich) supplemented with 10% vol/vol fetal bovine serum (Sigma‐Aldrich) and incubated in 37°C temperature and 5% CO_2_ conditions. For the immunoprecipitation experiments, dissociated cells were plated on 100 mm cell culture dishes (Corning). For immunofluorescent experiments, cells were plated at a density of 1 × 10^5^ cells on 20 mm coverslips precoated with poly‐l‐lysine (0.1 mg/ml; Sigma‐Aldrich).

### 2.4 DNA transfections and treatments

DNA plasmid vectors were transfected at 60%–90% cells' confluence with 2 mg/ml, pH 7.3, polyethylenimine HCl max solution (PEI max; Polysciences). Each cell culture dish, containing 3 × 10^6^ cells, received a DNA‐PEI max mixture consisting of 10 μg total DNA along with 20 μg PEI max in Opti‐MEM Reduced Serum Media (Thermo Fisher Scientific). After 48 h transfection, HEK293T cells were treated with 1–4 μM rapamycin (LC Laboratories), 5 μM AP21967 (Takara Bio) or 99% ethanol as a vehicle for 6–24 h before lysis or fixation.

### 2.5 Protein immunoprecipitation from HEK293T cells

HEK293T cells were lysed in ice‐cold lysis buffer (NaCl 150 mM, Tris–HCl 50 mM pH 7.4, EDTA 2 mM) containing 1% Triton X‐100, 0.1% sodium dodecyl sulfate, 10% glycerol, 1 × cOmplete Ultra tablets protease inhibitor cocktail (Roche), 1 mM sodium orthovanadate (Sigma‐Aldrich), 11 mM β‐glycerolphosphate, and 10 mM sodium fluoride (Sigma‐Aldrich). Each cell culture dish containing 3 × 10^6^ cells was treated with 1 ml lysis buffer with or without 2 μM rapamycin on ice, collected in a centrifuge tube, and spun down at 14,000 × *g,* 4°C. For each cell culture dish, 100 μl of supernatant were used as input and diluted with sodium dodecyl sulfate–polyacrylamide gel electrophoresis (SDS‐PAGE) sample buffer, and 900 μl of supernatant were used for immunoprecipitation with 10 μl of the anti‐Flag M2 antibody beads (Sigma‐Aldrich) for 2–4 h at 4°C. Beads were washed with 1 ml of lysis buffer three times. Bound proteins were eluted with SDS‐PAGE sample buffer or Flag‐peptide (Sigma‐Aldrich). Input and immunoprecipitated proteins were boiled for 5 min, and subjected to western blotting. *Spodoptera frugiperda*, *Sf*21 (baculovirus)‐derived BDNF protein (Bio‐Techne) was used to compare the biological activity of BDNF obtained from pro‐TEVcs‐BDNF.

### 2.6 In vitro TEV activity assay

For in vitro activity assay, purified Cerulean‐TEVcs‐YPet and purified constitutively active TEV or uniRapR‐TEV were diluted with a molar ratio of 1:1 in a reaction buffer consisting of: 50 mM Tri‐HCl pH 7.5, 0.5 mM EDTA, 1 mM DTT, 1 μM rapamycin. The reactions were performed at 30°C for 1, 6, and 24 h. Samples were then denatured at 95°C for 5 min and subjected to western blotting.

### 2.7 Western blotting

The supernatant was quantified for protein content (DC Protein Assay; Bio‐Rad). Equal amounts of protein were diluted in Laemmli buffer, boiled, and resolved by SDS‐PAGE. The primary antibodies (available in Table S4) were incubated from 1 h to overnight at 4°C and revealed with horseradish peroxidase‐conjugated secondary antibodies (Cell Signaling Technology). Expression was evaluated and documented by using UVItec Cambridge Alliance.

### 2.8 Immunofluorescence analysis

HEK293T cells transfected with Flag‐tagged proBDNF or pro‐TEVcs‐BDNF constructs were fixed with 4% paraformaldehyde for 20 min at room temperature (RT; 22–25°C), permeated with 0.1% Triton for 15 min prior to being blocked in 0.3% bovine serum albumin (BSA) for 20 min. Samples were incubated for 3 h with anti‐Flag (Sigma‐Aldrich) primary antibody diluted 1:200 in 0.3% BSA in phosphate‐buffered saline (PBS), washed twice in PBS and incubated with goat anti‐rabbit secondary antibody (Alexa Fluor 488; Thermo Fisher Scientific) diluted 1:1000 in PBS at RT for 90 min, light‐protected. Cell nuclei were counterstained with DAPI (1:1000 in PBS; Thermo Fisher Scientific) for 10 min at RT, light‐protected. Samples were coverslipped with an antifade medium (ProLong Gold; Thermo Fisher Scientific). Images were acquired by using a light microscope (Olympus BX63 microscope) equipped with ×10 and ×40 objective lens. Cell count and fluorescent‐intensity evaluation were performed by using ImageJ software by randomly selecting at least *n* = 4 fields for each condition.

### 2.9 Enzyme‐linked immunosorbent assay

ProBDNF and BDNF concentrations were determined by using commercially available ELISA kits (Immunological Sciences). The assays were performed according to the manufacturer's instructions.

### 2.10 Analysis of dendritic spines

Hippocampal organotypic slice (350 μm thickness) cultures were prepared from postnatal day 4–7 rats through a McIllwain tissue chopper. Gold particles coated with plasmids were biolistically discharged to slices at 6–8 days in culture (DIV) by using Gene‐Gun (Bio‐Rad) as previously described.^14^ BDNF‐SEP and TEV plasmids were transfected together with a plasmid encoding dsRed2 to identify the transfected cells. Particle‐mediated gene transfer can induce transgene expression both in astrocytes and neurons in an unpredictable manner at relatively distant areas.^15^ In our experiments, we selected transfected CA1 pyramidal neurons and studied morphology and the number of dendritic spines. Confocal microscopy images were acquired 3 days later, after being treated with either ethanol or 1 μM rapamycin for 1, 6, and 24 h. Images of transfected neurons with dsRed2 plasmids were acquired by using a confocal laser scanning system (Nikon Ti‐E; Confocal Head A1 MP) with a 60× oil‐immersion objective lens. Each experiment was repeated at least three times with independent neuronal preparations. DsRed2‐positive pyramidal neurons of the hippocampal CA1 region were identified by the presence of a basal dendritic tree, distinct single apical dendrite and dendritic spines. A researcher who was unaware of the identity of the specimens performed spine density and spine diameter evaluation in each neuron analyzed. The apical dendritic tree was examined and spine density was calculated along ~20 μm length of apical dendrite branches. Spines were characterized into classes based on the length and diameter of the head and neck. Specifically, a spine was labeled “thin” if its head was below 0.6 μm in diameter and had a maximal length that was at least twice as big as the head diameter. A spine was classified as a “mushroom” if its head diameter exceeded 0.6 μm.^16^ All analyses were performed by using Nikon Ti‐E software.

### 2.11 Stereotaxic injection

C57BL/6 mice of 2 months of age underwent stereotaxic surgery for cannulae implantation. Animals were anesthetized with a cocktail of ketamine (87.5 mg/kg) and xylazine (12.5 mg/kg) administered intraperitoneally. Each anesthetized mouse was placed in a stereotaxic apparatus (Stoelting Co), and the head skin was cut longitudinally. Pedestals with double fixed‐length guide cannulae (C235G‐3.0; Plastics One) directed at the dorsal hippocampus were attached to the calvarium with carboxylate cement (3M ESPE, Durelon; 3M Deutschland GmbH). Coordinates: posterior to bregma 2.1 mm; lateral to midline ±1.5 mm. One week later, animals underwent intracerebral BDNF injection. The animals were gently restrained and an infusion cannula connected to a microsyringe (10 μl; Hamilton) by a polyethylene tube was inserted into the guide cannula. A total volume of 1.5 μl (80 ng) of BDNF or vehicle (HBSS) was injected into each hippocampus at a flow rate of 0.5 μl/min. The infusion cannula was left in place for an additional 2 min at the end of the infusion to allow solution spreading. Thirty minutes after the injection, animals were sacrificed and the hippocampi were collected separately. In some animals, a solution of 4% methylene blue was infused for localization of infusion cannulas.

### 2.12 Statistical analysis

The statistical tests used (i.e., Student's *t*‐test, one‐way analysis of variance with either the Dunnett's or the Tukey's posthoc test comparisons) are indicated in in the corresponding figure legends for each experiment. All statistical tests were two‐tailed and the level of significance was set at 0.05. Results are shown as mean ± *SEM*.

### 2.13 Data availability

Data that support the finding of this study are available from the corresponding authors upon request.

## 3 RESULTS

### 3.1 Engineering an inducible single‐chain TEV protease

**FIGURE 1:**
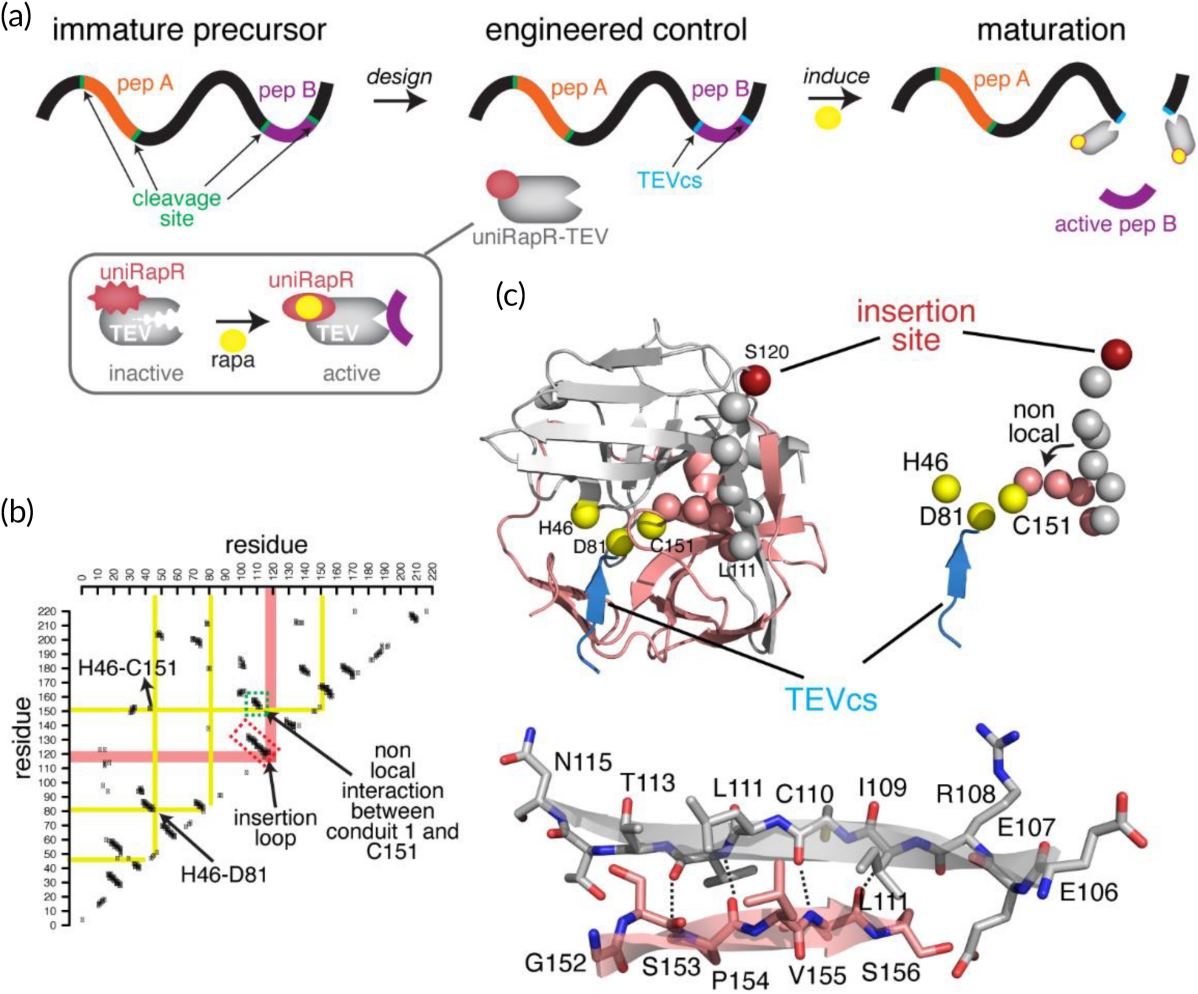
A strategy to control protein cleavage. (a) Our strategy involves the replacement of the endogenous cleavage sites with the TEVcs in the target premature precursor protein and the expression of engineered uniRapR‐TEV. Upon activation of uniRapR‐TEV, the active part of the peptide is released from the precursor. (b) Contact map indicating the surface‐exposed loops on TEV crystal structure, the loop that connects two closely interacting antiparallel β‐strands (E106‐N115 and S122‐V125), was selected as a putative insertion loop for the uniRapR domain. A contact is defined if Carbon‐α of two amino acids is within 5 Å. The contact clusters that are perpendicular to the diagonal graph line indicate the residues interacting on two structural units (β‐strands or α‐helices). One of such contact clusters, also named “conduits,” as one of which was shown in the dashed red rectangular box, represent the two interacting β‐strands (E106‐N115 and G152‐S157), which is connected by the putative insertion loop. The contacts between the selected conduit and the other regions of the protein are shown in salmon color. The yellow lines, which represent the important catalytic sites of TEV, intersecting with the salmon‐colored line indicate the nonlocal interactions. One such nonlocal interaction (green‐dashed box on the contact map) is between the β‐strands (E106‐N115) and the β‐strand (G152‐S157), where the first residue G152 of this strand is located next to C151, a site that is one of the three TEV catalytic triad residues along with H46 and D81. (c) Identified split insertion site (red sphere) is located on a surface‐exposed loop, shown on the crystal structure of wt TEV (PDB: 1LVM). TEVcs is shown as a blue β‐strand. The perturbation from the insertion site is transmitted to the catalytic triad via the β‐strands (gray spheres) connected by the insertion loop, and also the β‐strand that makes nonlocal contacts with another strand that elongates (salmon‐color spheres) to the triad residue C151. TEV, tobacco etch virus; TEVcs, TEV cleavage site; wt, wild‐type

The first step of the strategy using an engineered TEV is the replacement of the endogenous cleavage sites of target proteins with TEVcs (Figure 1a). We obtained an engineered allosteric control of TEV by inserting the uniRapR^17^ domain into an allosteric site that we had identified also based on our previous structural analysis^18^ (Figure 1b,c). When inserted at the surface exposed, evolutionarily nonconserved allosteric sites, the rapamycin‐unbound uniRapR domain induces a structural disorder both in uniRapR domain and in the host protein.^17^ Upon rapamycin binding, the uniRapR domain in the host protein becomes ordered and functional.^17^, ^18^, ^19^ Among the surface‐exposed loops on TEV crystal structure, the loop that connects two closely interacting antiparallel β‐strands (E106‐N115 and S122‐V125), as shown in the contact map (Figure 1b,c), was selected as a putative insertion loop for the uniRapR domain. On the basis of this structural analysis, we hypothesized that the putative insertion loop between N115‐M121 is mechanically coupled to the C151, thus perturbation at the allosteric site would perturb C151, thereby TEV catalytic activity. Based on this model, rapamycin‐unbound disordered uniRapR domain would dynamically perturb the β‐strands E106‐N115 and G152‐S157, and perturbed E106‐N115 strand would perturb the strand G152‐S156, which is next to C151. The perturbed C151 would inactivate TEV. Rapamycin binding would rescue all these native interactions and would rescue the activity of TEV.

**FIGURE 2:**
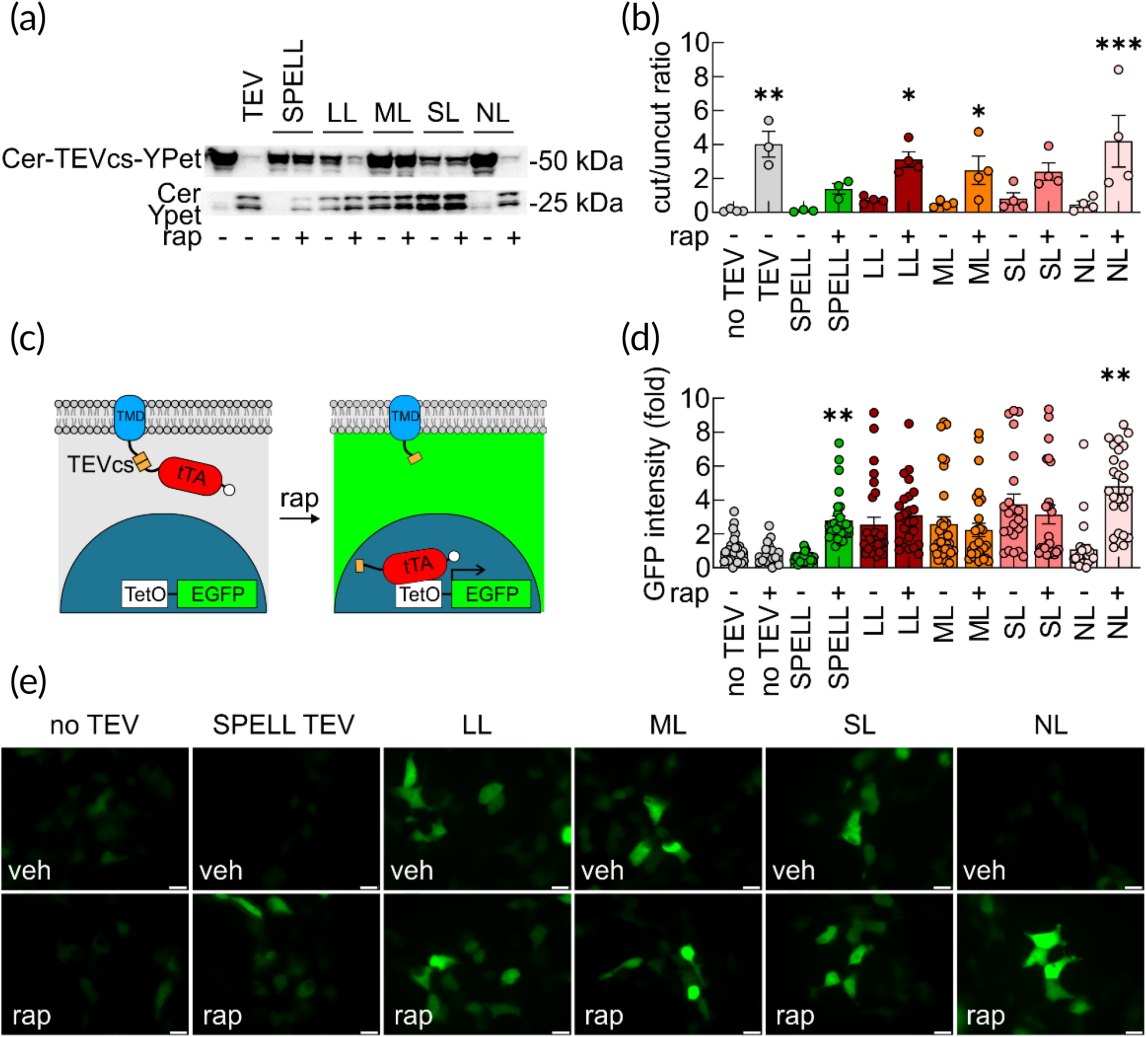
Assessment of uniRapR‐TEV designs. (a) Protease activity of constitutively active TEV, SPELL‐TEV, uniRapR‐TEV LL, uniRapR‐TEV ML, uniRapR‐TEV SL, uniRapR‐TEV NL, evaluated by monitoring the migration of the synthetic Cerulean‐TEVcs‐YPet protein cleavage in SDS‐PAGE experiments. LL, ML, SL, NL indicate long, medium, short, and no linker, respectively. HEK293T cells were treated with ethanol (veh) or 2 μM rapamycin (rap) for 6 h. (b) Summary graph of the ratio between densitometric values of the upper/lower bands representing the results of the Cerulean‐TEVcs‐YPet cleavage by TEV, SPELL‐TEV and uniRapR‐TEV analogs before and after addition of rap. Values are available in Table S1. Error bars represent *SEM* from three/four independent experiments. *p < 0.05, **p < 0.005, ***p < 0.0005 compared to no TEV condition; one‐way ANOVA with the Dunnett's posthoc test comparisons. (c) Schematic drawing of TMD‐TEVcs‐tTA in cells before and after TEV activity. TEV‐cleaved tTA translocates to the nucleus and triggers EGFP gene expression. Engineered TEVs (SPELL and uniRapR‐TEVs) cut TMD‐TEVcs‐tTA in the presence of rap. (d and e) Constitutive active TEV, SPELL‐TEV or uniRapR‐TEV constructs were transfected to HEK293T cells together with TMD‐TEVcs‐tTA and tetO‐EGFP. EGFP reporter gene expression was monitored in the presence of veh or 2 μM rap for 6 h by quantifying fluorescence intensity signal. No TEV means HEK293T cells transfected with TMD‐TEVcs‐tTA, tetO‐EGFP and an empty TEV vector. Values are available in Table S2. Error bars represent *SEM*. *p < 0.05, **p < 0.005 compared to No TEV condition; one‐way ANOVA with the Dunnett's posthoc test comparisons. Scale bar = 20 μm. ANOVA, analysis of variance; SDS‐PAGE, sodium dodecyl sulfate–polyacrylamide gel electrophoresis; TEV, tobacco etch virus; TEVcs, TEV cleavage site; TMD, transmembrane domain; tTA, tetracycline‐controlled transactivator

Because we aim to obtain minimum activity in the absence of rapamycin and maximum activity in the presence of rapamycin, as expected from an ideal engineered protein switch,^17^, ^18^, ^19^, ^20^, ^21^, ^22^ we used insertion linkers with different sizes to control the level of structural perturbation (Figure 2). The engineered constructs or control constructs were expressed in HEK293T cells with a TEV fluorescent substrate,^19^ which is the fusion of mCerulean and YPet proteins with TEVcs between them. This reporter was previously used to measure the resonance energy transfer from mCerulean to YPet for the engineering of the improved version of dual‐chain split TEV, that is, the split proteins regulated by a ligand or by light (SPELL) TEV.^19^ We first measured the protease activity of the engineered constructs by monitoring the SDS‐PAGE migration of Cerulean and YPet (Figure 2a,b). The previously reported split TEV^8^, ^10^, ^11^ demonstrated high activity in the absence of rapamycin (Figure S1), whereas the SPELL‐TEV^19^ showed negligible background activity in the absence of rapamycin (Figure 2a,b). Among our uniRapR‐TEV constructs, the one with no linker (NL) showed a similar almost undetectable background activity (Figure 2a,b). In the presence of rapamycin, NL uniRapR‐TEV construct exhibited almost full protease activity, even more than SPELL‐TEV (Figure 2a,b). The uniRapR‐TEV constructs with short (SL: G connecting amino acids on both sides of uniRapR), medium (ML: GGS connecting amino acids on both sides of uniRapR), and long (LL: GGSGGG connecting amino acids on both sides of uniRapR) linkers exhibited a substantial activity in the presence of rapamycin. However, for some constructs, we also observed background activity, similar to the response of the previously reported split TEV (Figures 2a,b and S1). To further test these constructs with an alternative approach, we have employed a system in which the induction of the cleavage can be measured with a fluorescent reporter expression, which is induced by the nuclear translocation of membrane‐localized transactivator that carries TEVcs (Figure 2c). We expressed our designs in HEK293T cells together with a tetracycline operator EGFP conjugated (tetO‐EGFP) and a synthetic protein consisting of a tetracycline‐controlled transactivator (tTA), linked to a transmembrane domain (TMD) through a TEVcs (TMD‐TEVcs‐tTA) (Figure 2c). Rapamycin‐mediated uniRapR‐TEV activation cleaved tTA that translocated to the nucleus and initiated EGFP expression (Figure 2c). Twenty‐four hours later, we observed a robust increase of EGFP expression in rapamycin‐treated HEK293T cells transfected with all uniRapR‐TEV constructs (Figure 2d,e). However, in control cells exposed to the vehicle, the EGFP signal was substantially lower in cells transfected with uniRapR‐TEV NL. These experiments showed that a switchable single‐chain TEV protease is useful to control protein cleavage in the cytosol. Finally, comparing the catalytic activity of purified uniRapR‐TEV with the purified constitutively active TEV in an in vitro assay using purified mCerulean‐TEVcs‐YPet as substrate, we found that engineered TEV maintains a similar catalytic kinetic of constitutively active TEV in substrate cleavage (Figure S1c).

### 3.2 Chemogenetic strategy for the control of protein cleavage in the secretory pathway

Given the large number of proteins cleaved by endogenous proteases in the secretory pathway rather than in cytosol, we sought out to design uniRapR‐TEV that can function in the secretory pathway as a chemogenetic strategy for the control of protein cleavage. Secreted proteolytic cleavage products are synthesized on the rough endoplasmic reticulum (ER) as pre‐proproteins.^23^, ^24^ The presequence peptide directs the proprotein synthesis to the rough ER where the presequence peptide is cleaved off immediately. Then the proproteins translocate from Golgi apparatus to the trans‐Golgi network (TGN), where the prodomain is cleaved off to yield the mature products. From the TGN, mature products can be either released continuously without any triggering stimulus or released in response to extracellular triggering events that elevate intracellular Ca^2+^ concentration.^23^, ^24^ For example, BDNF is continuously released from TGN with small vesicle granules in a Ca^2+^‐independent manner but it can also be released from larger vesicles upon Ca^2+^ influx induced by neuronal depolarization.^25^

**FIGURE 3:**
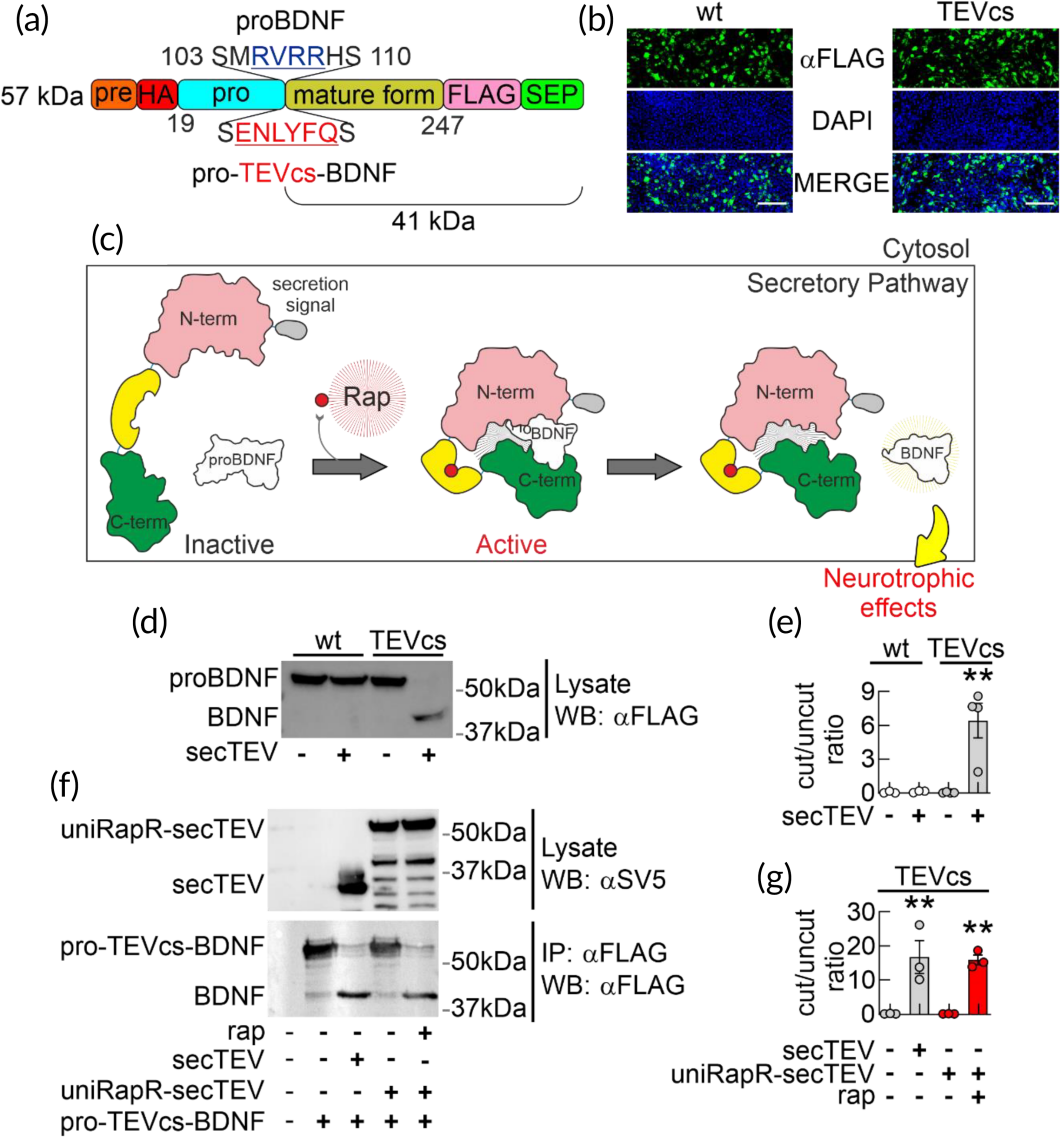
Testing uniRapR‐TEV to control pro‐TEVcs‐BDNF processing in living cells. (a) Schematic representation of the different domains of BDNF (predomain in orange; prodomain in blue; BDNF in gold) pointing out the wt sequence (MRVRR) recognized by endogenous proteases and the cleavage recognition sequence of TEV (ENLYFQS) inserted in the proBDNF to generate pro‐TEVcs‐BDNF. Different endogenous proteases (intracellular protein convertase, Furin, Plasmin) can cleave wt proBDNF recognizing MRVRR sequence. (b) Immunostaining of HEK293T cells expressing the Flag‐tagged wt proBDNF and mutant pro‐TEVcs‐BDNF. Transfected cells were immunostained with anti‐Flag (FITC, green) and with DAPI for nuclei (blue). Mutations introduced into proBDNF (from MRVRRH to ENLYFQ) did not alter the number of Flag‐positive cells. Scale bar = 100 μm. (c) Cartoon describing the rationale of the engineering approach we developed to control proBDNF cleavage and BDNF maturation in living cells. (d) Assessment of proBDNF cleavage by Western blot analysis of lysates from HEK293T cells transfected with a constitutively active secTEV along with either proBDNF or pro‐TEVcs‐BDNF. SecTEV cleaved pro‐TEVcs‐BDNF whereas it was ineffective on wt proBDNF. The molecular weight of both wt proBDNF and pro‐TEVcs‐BDNF is increased by Flag‐ and SEP‐tag at the C‐terminus and hemagglutinin (HA) tag between pre‐ and prodomains. (e) Summary of BDNF/proBDNF quantification ratio of (d). In HEK293T cells transfected with wt proBDNF alone, no evidence of overexpressed proBDNF cleavage was observed (0.06 ± 0.04, *n* = 3). Similarly, neither wt proBDNF in the presence of secTEV nor pro‐TEVcs‐BDNF alone produced bands corresponding to BDNF (0.07 ± 0.05, *n* = 3 and 0.04 ± 0.02, *n* = 4, respectively). In presence of secTEV, a significant increase of BDNF levels at the expense of pro‐TEVcs‐BDNF was observed (6.43 ± 1.53, *n* = 4). (f and g) Cleavage of pro‐TEVcs‐BDNF by uniRapR‐secTEV assessed by Western blot. HEK293T cells were cotransfected with pro‐TEVcs‐BDNF and either constitutively active secTEV or uniRapR‐secTEV. Blotting for Flag reveals disappearance of full‐length pro‐TEVcs‐BDNF and the appearance of BDNF in cells cotrasfected with constitutively active secTEV (16.70 ± 4.85 vs. 0.08 ± 0.05 in HEK293T cells transfected with pro‐TEVcs‐BDNF alone, *n* = 3) or uniRapR‐secTEV stimulated with 4 μM rap for 6 h (15.91 ± 1.43 vs. 0.06 ± 0.03 in HEK293T cells treated with EtOH, *n* = 3). Error bars represent *SEM*. **p < 0.005, compared to wt proBDNF alone (e) and pro‐TEVcs‐BDNF alone (g) conditions; one‐way ANOVA with the Dunnett's posthoc test comparisons. ANOVA, analysis of variance; BDNF, brain‐derived neurotrophic factor; TEV, tobacco etch virus; TEVcs, TEV cleavage site; wt, wild‐type

As a proof‐of‐concept, we used uniRapR‐TEV to control BDNF maturation (Figure 3). The BDNF is produced by proteolytic cleavage of proBDNF, catalyzed along the secretory pathway by Furin/proprotein convertase 1/3 (PC1/3) proteases,^26^, ^27^ and extracellularly through the tissue plasminogen activator/plasminogen cascade.^28^ By activating tropomyosin kinase‐B receptors (TrkB), BDNF plays a key role in synapse formation and maturation, synaptic plasticity, neural stem cell survival and differentiation.^25^, ^29^ Conversely, proBDNF can bind nerve growth factor receptors (NGFR also known as TNFRSF16 or p75 neurotrophin receptor), and it has been proposed to induce long‐term synaptic depression and apoptosis.^30^, ^31^

To make the BDNF maturation inducible, we first inserted TEVcs into the proBDNF sequence by replacing the Furin/Plasmin cleavage site^32^ (Figure 3a) that is highly conserved across mice, rats and humans (Figure S2). In Western blot experiments, the amount of this proBDNF containing the TEVcs (pro‐TEVcs‐BDNF) was similar to those of wild‐type (wt) proBDNF (Figures 3b,d and S3a), suggesting that this small sequence replacement does not block the protein production. We then evaluated the ability of uniRapR‐TEV to cleave pro‐TEVcs‐BDNF in a rapamycin‐dependent manner. As expected, neither uniRapR‐TEV in the presence of rapamycin, nor an active mutant form of TEV, cleaved pro‐TEVcs‐BDNF in living cells (i.e., HEK293T) because of the cytosolic localization of TEV proteases (Figure S3b,c). However, performing an in vitro protease assay after immunoprecipitation of both pro‐TEVcs‐BDNF and uniRapR‐TEV in the presence of rapamycin, we observed an SDS‐PAGE migration of the propeptide (Figure S3b,d,e). These data demonstrated the ability of uniRapR‐TEV to cut pro‐TEVcs‐BDNF, confirming that the cleavage in living cells depended on the localization of pro‐TEVcs‐BDNF and the engineered protease.

To make uniRapR‐TEV functionally active along the secretory pathway of mammalian cells, we added the secretion signal at the N‐terminus and introduced three mutations N23Q‐C130S‐T173G^13^ generating the uniRapR‐TEV variant targeting the secretory pathway (uniRapR‐secTEV) (Figures 3c and S4). The N23Q‐C130S‐T173G mutations were used to avoid *N*‐glycosylation and cysteine oxidation of TEV in the ER lumen, which may hamper the correct folding of the protein.^13^ The constitutively active secTEV^13^ cleaved pro‐TEVcs‐BDNF (Figure 3d,e). When HEK293T cells expressing uniRapR‐secTEV and pro‐TEVcs‐BDNF were treated with rapamycin, we observed BDNF maturation in living cells (Figures 3f,g and S5). This result demonstrates that uniRapR‐secTEV variant can be robustly activated by rapamycin within the secretory pathway.

### 3.3 Neurotrophic effects of inducible BDNF maturation in living neurons

**FIGURE 4:**
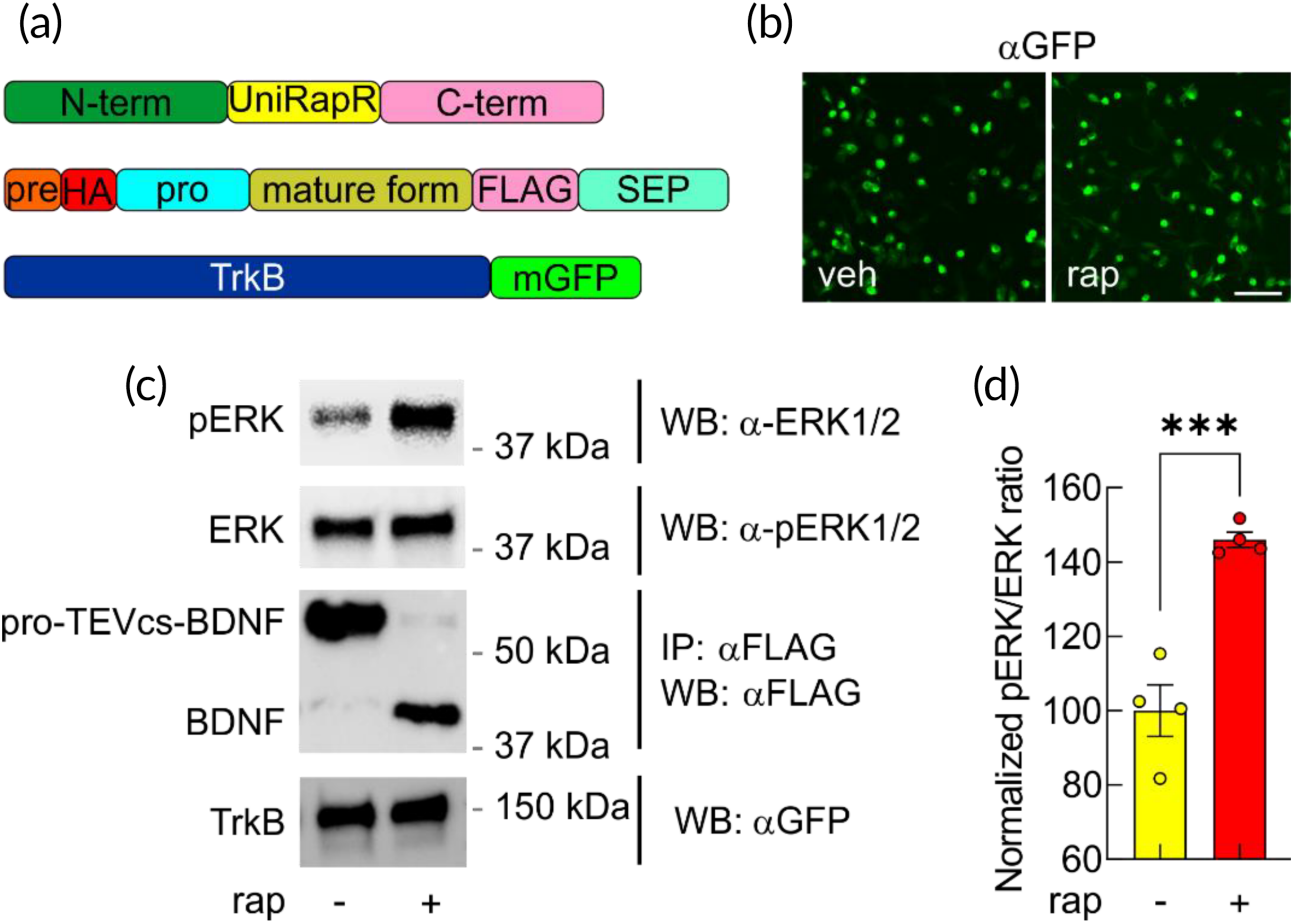
BDNF cleaved from pro‐TEVcs‐BDNF activates TrkB receptor‐mediated intracellular signals in living cells. (a) Schematic representation of plasmids used to transiently cotransfect HEK293T cells. (b) To examine the ability of BDNF cleaved from pro‐TEVcs‐BDNF to induce biological effects, HEK293T cells were transiently cotransfected with TrkB‐mGFP, pro‐TEVcs‐BDNF and uniRapR‐secTEV. Transfected cells were immunostained with anti‐GFP (FITC, green). Scale bar = 100 μm. (c) Representative western blots of lysates of HEK293T cells transfected with plasmids represented in (a) and treated with rapamycin or not (vehicle). (d) Pro‐TEVcs‐BDNF was cut by rapamycin‐mediated uniRapR‐secTEV activation, and BDNF induced a significant increase of ERK phosphorylation in living cells (100.00 ± 6.92, *n* = 4 vs. 145.99 ± 2.05, *n* = 4 in neurons treated with veh and rap, respectively). Error bars represent *SEM*. ***p < 0.0005 by Student's *t*‐test. BDNF, brain‐derived neurotrophic factor; ERK, extracellular signal‐regulated kinase; TEV, tobacco etch virus; TEVcs, TEV cleavage site; TrkB, tropomyosin kinase‐B receptor

**FIGURE 5:**
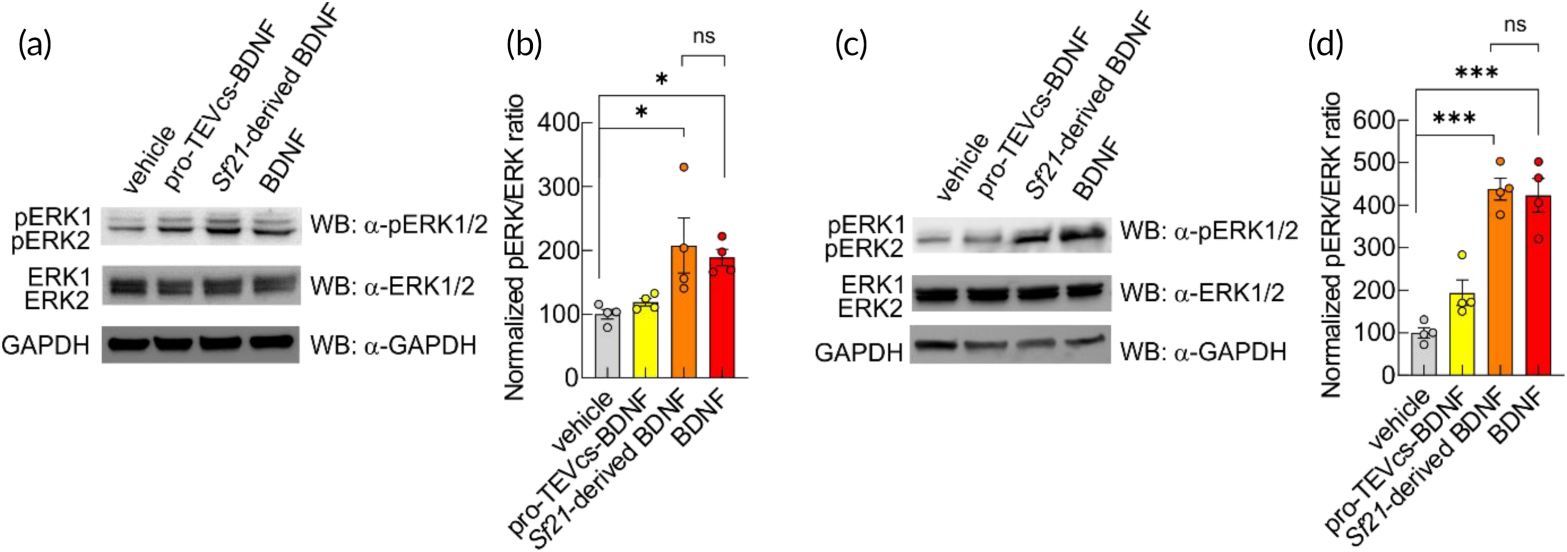
BDNF obtained from uniRapR‐secTEV promotes ERK1/2 phosphorylation indistinguishable from commercially available *Sf21*‐derived BDNF. (a) Representative western blots of the BDNF‐induced ERK signaling in HEK293T cells transiently expressing TrkB and stimulated with purified pro‐TEVcs‐BDNF (10 ng/ml) or *Sf21*‐derived BDNF (10 ng/ml) or BDNF purified from pro‐TEVcs‐BDNF (10 ng/ml) for 30 min. Cell lysates were collected for SDS‐PAGE. Western blot analysis showed that p‐ERK/ERK was significantly increased after both *Sf21*‐derived BDNF and BDNF stimulation. Anti‐glyceraldehyde‐3‐phosphate dehydrogenase (GAPDH) was used as an internal control. (b) Cells treated with either vehicle or pro‐TEVcs‐BDNF showed significantly lower activation of ERK signaling (vehicle: 100.00 ± 7.56%, *n* = 4; pro‐TEVcs‐BDNF: 118.90 ± 5.83%, *n* = 4; *Sf21*‐derived BDNF: 207.50 ± 43.13%, *n* = 4; BDNF: 188.70 ± 13.19%, *n* = 4). (c) Representative western blots of the BDNF‐induced ERK signaling in organotypic hippocampal slice cultures. Organotypic hippocampal slice cultures were stimulated with purified pro‐TEVcs‐BDNF (50 ng/ml) or *Sf21*‐derived BDNF (50 ng/ml) or BDNF (50 ng/ml) for 30 min. Cell lysates were collected for SDS‐PAGE. Western blot analysis showed that p‐ERK/ERK was significantly increased after BDNF stimulation. (d) In slice cultures treated with either vehicle or pro‐TEVcs‐BDNF activation of ERK signaling was significantly lower than in slice cultures treated with BDNF (vehicle: 100.00 ± 11.97%, *n* = 4; pro‐TEVcs‐BDNF: 194.00 ± 30.09%, *n* = 4; *Sf21*‐derived BDNF: 437.80 ± 25.77%, *n* = 4; BDNF: 423.20 ± 39.55%, *n* = 4). Anti‐GAPDH was used as an internal control. Error bars represent *SEM*. ns, not significant, *p < 0.05, ***p < 0.0005 compared to vehicle condition; one‐way ANOVA with the Tukey's posthoc test comparisons. BDNF, brain‐derived neurotrophic factor; ERK, extracellular signal‐regulated kinase; SDS‐PAGE, sodium dodecyl sulfate–polyacrylamide gel electrophoresis; TEV, tobacco etch virus; TEVcs, TEV cleavage site; TrkB, tropomyosin kinase‐B receptor

To assess whether synthetically maturated BDNF can bind and activate its target receptor TrkB,^33^ we expressed pro‐TEVcs‐BDNF, uniRapR‐secTEV with TrkB‐mGFP. Chemogenetic activation of uniRapR‐secTEV led to a clear increase of ERK phosphorylation (Figure 4). To further analyze and compare the biological activity of the chemogenetically maturated BDNF with a commercially available *Sf21*‐derived BDNF, we immunoprecipitated, purified and quantified pro‐TEVcs‐BDNF or BDNF from HEK293T cells cotrasfected with uniRapR‐secTEV and treated with vehicle or rapamycin (Figure S5). Subsequently, both HEK293T cells expressing TrkB and organotypic hippocampal slices treated for 30 min with: (i) chemogenetically maturated BDNF or (ii) the commercially available *Sf21*‐derived BDNF showed a significant increase in ERK phosphorylation signals without a significant difference between the effects induced by the two different BDNF (Figure 5a–d). These data demonstrated that the minimal perturbation introduced in proBDNF did not affect the biological activity of BDNF.

The advantages of the system proposed here include tight regulation of TEV in subcellular compartments and the availability of membrane‐permeable nonimmunosuppressive analogs of rapamycin. Thus, in a set of experiments, we evaluated the ability of nonimmunosuppressive rapamycin analogs in promoting uniRapR‐secTEV activation. As expected uniRapR‐secTEV was activated by AP21967 similarly to rapamycin (Figure S6), further supporting a potential translational application of our chemogenetic strategy.

**FIGURE 6:**
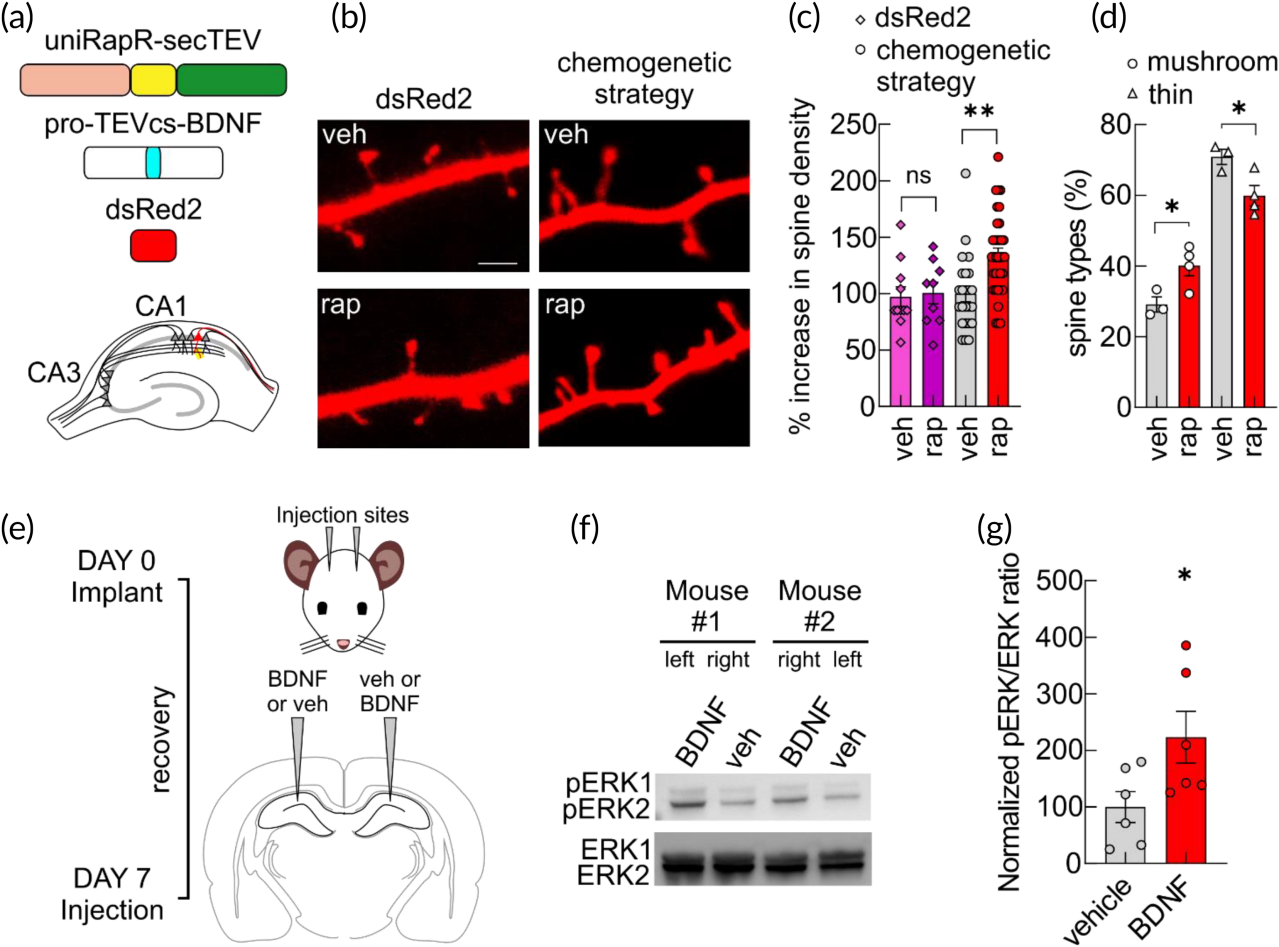
BDNF maturation obtained from uniRapR‐secTEV promotes dendritic spines changes in living neurons and BDNF signaling in in vivo mouse brain. (a) Schematic diagram showing the constructs used to biolistically transfect slice cultures. (b) Representative confocal images of dendritic spines in neurons of organotypic slices transfected with dsRed2 or with our chemogenetic strategy (pro‐TEVcs‐BDNF, uniRapR‐secTEV, and dsRed2) and treated with either EtOH (veh) or 1 μM rap for 24 h. Scale bar = 2 μm. (c) For neurons transfected with dsRed2: % increase in spine density (97.32 ± 9.19, *n* = 11 vs. 100.60 ± 10.16, *n* = 9 in neurons treated with veh and rap, respectively); for neurons transfected with chemogenetic strategy: % increase in spine density (100.00 ± 7.21, *n* = 23 vs. 134.40 ± 6.01, *n* = 35 in neurons treated with veh and rap, respectively). In each experiment, at least three segments (20 μm) from secondary dendrites from two neurons were analyzed. (d) Percent distribution of spine types (mushroom spines: 29.14 ± 2.14% vs. 40.11 ± 2.88%, in neurons treated with veh and rap, respectively; thin spines: 70.86 ± 2.14% vs. 59.88 ± 2.88%, in neurons treated with veh and rap, respectively). At least three independent experiments were performed. Error bars indicate *SEM*. ns, not significant, *p < 0.05, **p < 0.005 compared to vehicle condition; one‐way ANOVA with the Dunnett's posthoc test comparisons. (e) Schematic representation of the experimental design and time schedule of the protocol. (f) Representative western blots of the BDNF‐induced ERK signaling in two mice injected with BDNF (left and right hemisphere in mouse #1 and #2, respectively) and vehicle (right and left hemisphere in mouse #1 and #2, respectively). (g) BDNF induced a significant increase of ERK phosphorylation in in vivo mouse hippocampi (% increase 100.00 ± 27.43, *n* = 6 vs. 223.20 ± 45.68, *n* = 6 in hemispheres injected with veh and BDNF, respectively). *p < 0.05 by Student's *t*‐test. ANOVA, analysis of variance; BDNF, brain‐derived neurotrophic factor; ERK, extracellular signal‐regulated kinase; TEV, tobacco etch virus; TEVcs, TEV cleavage site

Although it has been shown that BDNF supports a variety of dendritic spine functions, including spine maturation and plasticity, the exact contribution of BDNF secreted from pre‐ versus postsynaptic sites is still under debate. A presynaptic release of BDNF contributes to its paracrine actions, whereas its postsynaptic secretion has been suggested to be responsible for the autocrine effects.^25^, ^34^, ^35^ Indeed, the biochemical characteristics of BDNF, including the positive charges on its surface, prevent its diffusion and keep its action locally at synapses.^36^ This feature also hampers the effectiveness of purified BDNF injections as a therapeutic strategy. Because extracellular application could affect both sides, the exogenous application of purified BDNF is also not useful to dissect the roles of pre‐ versus postsynaptic release. However, the genetic deletion of BDNF, specifically in CA3 or CA1 hippocampal neurons, revealed that the paracrine release affects the strength of synaptic plasticity, whereas autocrine BDNF signaling contributes to the maintenance of synaptic potentiation.^37^ These genetic approaches carry the deletion of both proBDNF and BDNF, thus the contribution of each form to the observed effect is not distinguishable. The chemogenetic strategy we have developed here to control protein cleavage should also be useful to evaluate the specific autocrine action of inducible intracellular BDNF maturation in hippocampal CA1 pyramidal neurons. To monitor the specific effects of BDNF in postsynaptic neurons, we ballistically transfected organotypic hippocampal slices with uniRapR‐secTEV, pro‐TEVcs‐BDNF, and dsRed2 to fluorescently label the transfected cells (Figure 6). After 48 h, we activated uniRapR‐secTEV with rapamycin for 24 h. We observed a significant increase in dendritic spine density and an increased volume of spine heads in hippocampal CA1 pyramidal neurons (Figures  6a‐c and S7) along with significant changes in spine morphology (Figure 6d). Interestingly, we found that activation of uniRapR‐secTEV with rapamycin for 1 h was sufficient to generate a significant increase in dendritic spine density after 24 h of the beginning of the treatment (Figure S8). Moreover, control experiments were performed to exclude an effect of rapamycin in dendritic spine density in hippocampal CA1 pyramidal neurons transfected with dsRed2 alone and no difference in dendritic spine number was observed comparing vehicle‐ versus rapamycin‐treated neurons (Figure 6a–d). These data showed that the spontaneous release of BDNF induced an autocrine trophic effect in hippocampal CA1 pyramidal neurons independently of synaptic plasticity induction (Figures 6 and S7). To further support the potential translational application of our system, we assessed whether intrahippocampal injections of chemogenetically‐produced BDNF were able to promote BDNF signaling in vivo. Thirty minutes after the injection, we found that ERK phosphorylation was increased in the hippocampi of hemispheres treated with chemogenetically‐produced BDNF compared with hippocampi of hemispheres treated with vehicle (Figure 6e–g). We thus generated a strategy enabling a complete cleavage of protein of interest in subcellular compartments, which allowed us to create and test inducible proteolytic cleavage products.

## 4 DISCUSSION

Many biological processes use proteases to activate or inactivate cellular signaling cascades, thus inducible proteases are crucial to address physiological and pathophysiological questions as well as to open the way for the development of new therapeutic approaches. Here, we introduce a new genetically encoded engineered single‐chain TEV allowing to control proprotein cleavage in different compartments of living mammalian cells leading to protein maturation. Our approach relies on NIa TEV protease. This protein is highly sequence‐specific, stable at broad pH and temperature ranges, and exhibits negligible activity toward endogenous mammalian proteomes rendering its expression tolerable for a broad range of organisms.^8^, ^9^, ^10^, ^11^, ^13^, ^38^ These remarkable characteristics of TEV are exploited in various protein engineering fields.^7^, ^8^, ^9^, ^10^, ^11^, ^39^, ^40^ To date, the approaches used to chemically control TEV proteolytic activity consisted of coexpression of N‐ and C‐inactive TEV fragments linked with dimerization domains.^8^, ^9^, ^10^, ^11^ The recent development of genetically encoded dual‐protein switch systems^39^, ^40^ overcame the background activity of split TEV by masking the TEVcs with a Jα‐helix of *Avena sativa* phototropin 1 light‐oxygen‐voltage 2 domains (AsLOV2)^41^ released only upon blue light exposure. Moreover, to improve the signal‐to‐background ratio, the low‐affinity sequence ENLYFQ/M into TEV substrates was used rather than the high‐affinity TEV sequence ENLYFQ/S.^7^ Of note, the new genetically encoded switchable single‐chain TEV proposed in this paper allows obtaining a chemically inducible cleavage of proteins of interest in different compartments of living mammalian cells overcoming the limits of previous chemically inducible TEV. Here, we demonstrated a set of controllable proteolytic effects, including cytosolic synthetic protein cleavage (Figure 2a,b), the inducible release of transactivation of gene expression (Figure 2c–e), and maturation of endogenous neurotrophins (e.g., BDNF), thus showing the versatility of this technique (Figures 3, 4, 5, 6, S5, S6 and S7). Our strategy is based on the insertion of TEVcs in the desired cutting point or the replacement of the endogenous cleavage site with the TEVcs. As a proof‐of‐concept, we replaced the endogenous, conserved MRVRRH sequence of proBDNF with ENLYFQ cleavage sequence generating a proBDNF analog pro‐TEVcs‐BDNF, whose protein amount was similar to wt proBDNF in transfected HEK293T cells. Coexpression of uniRapR‐TEV and pro‐TEVcs‐BDNF allowed a controlled BDNF maturation in both HEK293T cells and hippocampal CA1 pyramidal neurons. BDNF produced with the strategy we developed was biologically active, given that it was able to induce TrkB activation and ERK1/2 phosphorylation (Figures 4, 5, 6). Interestingly enough, in hippocampal CA1 pyramidal neurons, BDNF obtained with our strategy affected dendritic spines, the site of excitatory input onto neurons mediating synaptic transmission and plasticity.^42^ Specifically, we found that BDNF maturation induced spinogenesis, with a significant increase in spine density and volume as well as in the mushroom to thin spine ratio (Figure 6a‐d). Thus, our data indicate that the action of endogenous proteases is a critical step in the BDNF biology because once mature, BDNF promotes increment in dendritic spine number and a significant increase in the percentage of mushroom spines together with a significant decrease of thin spines (Figure 6a‐d). The mushroom spines have been suggested to be sites of long‐term memory storage,^43^, ^44^ whereas the thin spines undergo substantial structural plasticity, a critical phenomenon for the formation of new memories.^43^, ^44^ Of note, major brain pathologies, including autism spectrum disorders, schizophrenia, and Alzheimer's disease are characterized by marked disruptions of information processing and cognition, and recent evidence implicates dendritic spines as important substrates of these disorders' pathogenesis.^45^

Given the therapeutic potential of BDNF and the development of nonimmunosuppressive analogs of rapamycin,^3^, ^4^, ^46^ the biotechnological tools we are proposing here might also be applicable in vivo to overcome the well‐known limitations of standard approaches based on the injection of purified BDNF or *Bdnf* gene overexpression.^3^, ^4^ Indeed, despite the encouraging amount of preclinical data on the therapeutic use of BDNF, the ability to deliver neurotrophins to the brain still remains a challenge.^47^ Although clinical studies have demonstrated the presence of BDNF in cerebrospinal fluid following intrathecal administration of recombinant BDNF, no significant effects have been observed in counteracting or slowing the course of neurodegenerative diseases following exogenous treatment of BDNF.^48^, ^49^ However, some patients treated with high BDNF doses experienced sleep and behavioral disturbances.^50^ For these reasons, in recent years, new approaches have been sought to optimally and controllable convey BDNF in the CNS, such as, for example, neural stem cell transplantation or nanoparticle‐mediated therapies.^49^, ^51^

The absence of TEV sequence in mammalian proteome together with the high sequence stringency exhibited by TEV,^7^, ^13^ render our system a potential strategy to control protein cleavage in vivo without significantly interfering with cell viability. Of note, our data demonstrated that hippocampal CA1 pyramidal neurons transfected with uniRapR‐TEV and pro‐TEVcs‐BDNF were viable and showed spine density not significantly different from hippocampal CA1 pyramidal neurons transfected with dsRed2 only (Figure 6), indicating that uniRapR‐secTEV is well‐tolerated by living mammalian cells. Moreover, the absence of detrimental effects on dendritic spines in neurons overexpressing pro‐TEVcs‐BDNF could be in line with the observations that proBDNF receptors p75 are highly expressed in the first postnatal week with levels dropping at later ages.^52^ However, for future in vivo applications, to overcome potential side effects, it will be possible to transfer uniRapR‐secTEV and pro‐TEVcs‐BDNF constructs in viral vectors equipped with inducible gene expression systems to promote engineered protein synthesis in short periods of time.^53^, ^54^ Thus, in combination with specific promoters, our switchable strategy might be used to control proproteins maturation in subcellular compartments of targeted neurons of specific brain areas and for certain time windows.

### 4.1 Conclusion

The findings presented here show that uniRapR‐TEV is a novel tool with proven ability to chemically control the cleavage of proteins in either the cytosol or the secretory pathway through the interaction with rapamycin and its nonimmunosuppressive analogs, which are both permeable to the plasma membrane and secretory pathway. The novel chemogenetic single‐chain TEV reported here should be used for maturation, activation or inactivation of key regulatory proteins to address important physiological and pathophysiological questions as well as for the development of new gene therapy strategies.

## AUTHOR CONTRIBUTIONS

**Pietro Renna:** Data curation (equal); formal analysis (equal); investigation (lead). **Cristian Ripoli:** Conceptualization (equal); funding acquisition (equal); investigation (equal); project administration (equal); writing – original draft (equal); writing – review and editing (equal). **Onur Dagliyan:** Conceptualization (equal); methodology (equal); writing – original draft (equal). **Francesco Pastore:** Investigation (supporting). **Marco Rinaudo:** Investigation (supporting). **Agnese Re:** Investigation (equal). **Fabiola Paciello:** Investigation (equal). **Claudio Grassi:** Conceptualization (equal); funding acquisition (equal); writing – original draft (equal); writing – review and editing (equal).

## CONFLICT OF INTERESTS

A patent application has been submitted for the biotechnologies reported in this article: Cristian Ripoli, Pietro Renna, and Claudio Grassi. Application No. 102020000018064 and PCT/IB2021/056788 (Università Cattolica del Sacro Cuore and Fondazione Policlinico Universitario A. Gemelli IRCCS). The remaining authors declare no competing interests.

### PEER REVIEW

The peer review history for this article is available at https://publons.com/publon/10.1002/btm2.10292.

## Supporting information


**Appendix** S1: Supporting Information

## Data Availability

Data that support the finding of this study are available from the corresponding authors upon request.

## References

[1] Puente XS , Sánchez LM , Overall CM , López‐Otín C . Human and mouse proteases: a comparative genomic approach. Nat Rev Genet. 2003;4:544‐558. doi:10.1038/nrg1111 12838346

[2] Merighi A , Salio C , Ferrini F , Lossi L . Neuromodulatory function of neuropeptides in the normal CNS. J Chem Neuroanat. 2011;42:276‐287. doi:10.1016/j.jchemneu.2011.02.001 21385606

[3] Lu B , Nagappan G , Guan X , Nathan PJ , Wren P . BDNF‐based synaptic repair as a disease‐modifying strategy for neurodegenerative diseases. Nat Rev Neurosci. 2013;14:401‐416. doi:10.1038/nrn3505 23674053

[4] Nagahara AH , Tuszynski MH . Potential therapeutic uses of BDNF in neurological and psychiatric disorders. Nat Rev Drug Discov. 2011;10:209‐219. doi:10.1038/nrd3366 21358740

[5] Chung HK , Lin MZ . On the cutting edge: protease‐based methods for sensing and controlling cell biology. Nat Methods. 2020;17:885‐896. doi:10.1038/s41592-020-0891-z 32661424

[6] Ward OP . Proteases. In: Moo‐Young M , ed. Comprehensive Biotechnology. Pergamon; 2011:604‐615. doi:10.1016/B978-0-444-64046-8.00187-7

[7] Sanchez MI , Ting AY . Directed evolution improves the catalytic efficiency of TEV protease. Nat Methods. 2020;17:167‐174. doi:10.1038/s41592-019-0665-7 31819267 PMC7004888

[8] Gray DC , Mahrus S , Wells JA . Activation of specific apoptotic caspases with an engineered small‐molecule‐activated protease. Cell. 2010;142:637‐646. doi:10.1016/j.cell.2010.07.014 20723762 PMC3689538

[9] Wehr MC , Laage R , Bolz U , et al. Monitoring regulated protein‐protein interactions using split TEV. Nat Methods. 2006;3:985‐993. doi:10.1038/nmeth967 17072307

[10] Wintgens JP , Rossner MJ , Wehr MC . Characterizing dynamic protein‐protein interactions using the genetically encoded split biosensor assay technique split TEV. Methods Mol Biol. 2017;1596:219‐238. doi:10.1007/978-1-4939-6940-1_14 28293890

[11] Williams DJ , Puhl HL 3rd , Ikeda SR . Rapid modification of proteins using a rapamycin‐inducible tobacco etch virus protease system. PLoS One. 2009;4:e7474. doi:10.1371/journal.pone.0007474 19830250 PMC2760398

[12] Kapust RB , Tözsér J , Fox JD , et al. Tobacco etch virus protease: mechanism of autolysis and rational design of stable mutants with wild‐type catalytic proficiency. Protein Eng. 2001;14(12):993‐1000. doi:10.1093/protein/14.12.993 11809930

[13] Cesaratto F , López‐Requena A , Burrone OR , Petris G . Engineered tobacco etch virus (TEV) protease active in the secretory pathway of mammalian cells. J Biotechnol. 2015;212:159‐166. doi:10.1016/j.jbiotec.2015.08.026 26327323

[14] Spinelli M , Fusco S , Mainardi M , et al. Brain insulin resistance impairs hippocampal synaptic plasticity and memory by increasing GluA1 palmitoylation through FoxO3a. Nat Commun. 2017;8:2009. doi:10.1038/s41467-017-02221-9 29222408 PMC5722929

[15] Benediktsson AM , Schachtele SJ , Green SH , Dailey ME . Ballistic labeling and dynamic imaging of astrocytes in organotypic hippocampal slice cultures. J Neurosci Methods. 2005;141:41‐53. doi:10.1016/j.jneumeth.2004.05.013 15585287

[16] Paciello F , Podda MV , Rolesi R , et al. Anodal transcranial direct current stimulation affects auditory cortex plasticity in normal‐hearing and noise‐exposed rats. Brain Stimul. 2018;11:1008‐1023. doi:10.1016/j.brs.2018.05.017 29929771

[17] Dagliyan O , Shirvanyants D , Karginov AV , et al. Rational design of a ligand‐controlled protein conformational switch. Proc Natl Acad Sci U S A. 2013;110:6800‐6804. doi:10.1073/pnas.1218319110 23569285 PMC3637791

[18] Dagliyan O , Tarnawski M , Chu PH , et al. Engineering extrinsic disorder to control protein activity in living cells. Science. 2016;354:1441‐1444. doi:10.1126/science.aah3404 27980211 PMC5362825

[19] Dagliyan O , Krokhotin A , Ozkan‐Dagliyan I , et al. Computational design of chemogenetic and optogenetic split proteins. Nat Commun. 2018;9:4042. doi:10.1038/s41467-018-06531-4 30279442 PMC6168510

[20] Dagliyan O , Dokholyan NV , Hahn KM . Engineering proteins for allosteric control by light or ligands. Nat Protoc. 2019;14:1863‐1883. doi:10.1038/s41596-019-0165-3 31076662 PMC6648709

[21] Dagliyan O , Karginov AV , Yagishita S , et al. Engineering Pak1 allosteric switches. ACS Synth Biol. 2017;6:1257‐1262. doi:10.1021/acssynbio.6b00359 28365983 PMC5562282

[22] Dagliyan O , Hahn KM . Controlling protein conformation with light. Curr Opin Struct Biol. 2019;57:17‐22. doi:10.1016/j.sbi.2019.01.012 30849716 PMC6697613

[23] Al‐Qudah MA , Al‐Dwairi A . Mechanisms and regulation of neurotrophin synthesis and secretion. Neurosciences. 2016;21:306‐313. doi:10.17712/nsj.2016.4.20160080 27744458 PMC5224427

[24] Rafieva LM , Gasanov EV . Neurotrophin Propeptides: biological functions and molecular mechanisms. Curr Protein Pept Sci. 2016;17:298‐305. doi:10.2174/1389203716666150623104145 26100281

[25] Zagrebelsky M , Tacke C , Korte M . BDNF signaling during the lifetime of dendritic spines. Cell Tissue Res. 2020;382:185‐199. doi:10.1007/s00441-020-03226-5 32537724 PMC7529616

[26] Lee R , Kermani P , Teng KK , Hempstead BL . Regulation of cell survival by secreted proneurotrophins. Science. 2001;294:1945‐1948. doi:10.1126/science.1065057 11729324

[27] Zhang XY , Liu F , Chen Y , Guo WC , Zhang ZH . Proprotein convertase 1/3‐mediated down‐regulation of brain‐derived neurotrophic factor in cortical neurons induced by oxygen‐glucose deprivation. Neural Regen Res. 2020;15:1066‐1070. doi:10.4103/1673-5374.270314 31823886 PMC7034267

[28] Pang PT , Teng HK , Zaitsev E , et al. Cleavage of proBDNF by tPA/plasmin is essential for long‐term hippocampal plasticity. Science. 2004;306:487‐491. doi:10.1126/science.1100135 15486301

[29] De Vincenti AP , Ríos AS , Paratcha G , Ledda F . Mechanisms that modulate and diversify BDNF functions: implications for hippocampal synaptic plasticity. Front Cell Neurosci. 2019;13:135. doi:10.3389/fncel.2019.00135 31024262 PMC6465932

[30] Teng HK , Teng KK , Lee R , et al. ProBDNF induces neuronal apoptosis via activation of a receptor complex of p75NTR and sortilin. J Neurosci. 2005;25:5455‐5463. doi:10.1523/JNEUROSCI.5123-04.2005 15930396 PMC6724992

[31] Yang J , Harte‐Hargrove LC , Siao CJ , et al. proBDNF negatively regulates neuronal remodeling, synaptic transmission, and synaptic plasticity in hippocampus. Cell Rep. 2014;7:796‐806. doi:10.1016/j.celrep.2014.03.040 24746813 PMC4118923

[32] Koshimizu H , Kiyosue K , Hara T , et al. Multiple functions of precursor BDNF to CNS neurons: negative regulation of neurite growth, spine formation and cell survival. Mol Brain. 2009;2:27. doi:10.1186/1756-6606-2-27 19674479 PMC2743674

[33] Guo W , Nagappan G , Lu B . Differential effects of transient and sustained activation of BDNF‐TrkB signaling. Dev Neurobiol. 2018;78:647‐659. doi:10.1002/dneu.22592 29575722

[34] Harward SC , Hedrick NG , Hall CE , et al. Autocrine BDNF‐TrkB signalling within a single dendritic spine. Nature. 2016;538:99‐103. doi:10.1038/nature19766 27680698 PMC5398094

[35] Hedrick NG , Harward SC , Hall CE , Murakoshi H , McNamara JO , Yasuda R . Rho GTPase complementation underlies BDNF‐dependent homo‐ and heterosynaptic plasticity. Nature. 2016;538:104‐108. doi:10.1038/nature19784 27680697 PMC5361895

[36] Horch HW , Katz LC . BDNF release from single cells elicits local dendritic growth in nearby neurons. Nat Neurosci. 2002;5:1177‐1184. doi:10.1038/nn927 12368805

[37] Lin PY , Kavalali ET , Monteggia LM . Genetic dissection of presynaptic and postsynaptic BDNF‐TrkB Signaling in synaptic efficacy of CA3‐CA1 synapses. Cell Rep. 2018;24:1550‐1561. doi:10.1016/j.celrep.2018.07.020 30089265 PMC7176480

[38] Cesaratto F , Burrone OR , Petris G . Tobacco etch virus protease: a shortcut across biotechnologies. J Biotechnol. 2016;231:239‐249. doi:10.1016/j.jbiotec.2016.06.012 27312702

[39] Lee D , Hyun JH , Jung K , Hannan P , Kwon HB . A calcium‐ and light‐gated switch to induce gene expression in activated neurons. Nat Biotechnol. 2017;35:858‐863. doi:10.1038/nbt.3902 28650460

[40] Wang W , Wildes CP , Pattarabanjird T , et al. A light‐ and calcium‐gated transcription factor for imaging and manipulating activated neurons. Nat Biotechnol. 2017;35:864‐871. doi:10.1038/nbt.3909 28650461 PMC5595644

[41] Wu YI , Frey D , Lungu OI , et al. A genetically encoded photoactivatable Rac controls the motility of living cells. Nature. 2009;461:104‐108. doi:10.1038/nature08241 19693014 PMC2766670

[42] Harnett MT , Makara JK , Spruston N , Kath WL , Magee JC . Synaptic amplification by dendritic spines enhances input cooperativity. Nature. 2012;491:599‐602. doi:10.1038/nature11554 23103868 PMC3504647

[43] Bourne J , Harris KM . Do thin spines learn to be mushroom spines that remember? Curr Opin Neurobiol. 2007;17:381‐386. doi:10.1016/j.conb.2007.04.009 17498943

[44] Hayashi Y , Majewska AK . Dendritic spine geometry: functional implication and regulation. Neuron. 2005;46:529‐532. doi:10.1016/j.neuron.2005.05.006 15944122

[45] Penzes P , Cahill ME , Jones KA , VanLeeuwen JE , Woolfrey KM . Dendritic spine pathology in neuropsychiatric disorders. Nat Neurosci. 2011;14:285‐293. doi:10.1038/nn.2741 21346746 PMC3530413

[46] Vogel R , Mammeri H , Mallet J . Lentiviral vectors mediate nonimmunosuppressive rapamycin analog‐induced production of secreted therapeutic factors in the brain: regulation at the level of transcription and exocytosis. Hum Gene Ther. 2008;19:167‐178. doi:10.1089/hum.2007.125 18179357

[47] Zuccato C , Cattaneo E . Brain‐derived neurotrophic factor in neurodegenerative diseases. Nat Rev Neurol. 2009;5:311‐322. doi:10.1038/nrneurol.2009.54 19498435

[48] A controlled trial of recombinant methionyl human BDNF in ALS: the BDNF study group (phase III). Neurology. 1999;52:1427‐1433. doi:10.1212/wnl.52.7.1427 10227630

[49] Deng P , Anderson JD , Yu AS , Annett G , Fink KD , Nolta JA . Engineered BDNF producing cells as a potential treatment for neurologic disease. Expert Opin Biol Ther. 2016;16:1025‐1033. doi:10.1080/14712598.2016.1183641 27159050 PMC5762114

[50] Ochs G , Penn RD , York M , et al. A phase I/II trial of recombinant methionyl human brain derived neurotrophic factor administered by intrathecal infusion to patients with amyotrophic lateral sclerosis. Amyotroph Lateral Scler Other Motor Neuron Disord. 2000;1:201‐206. doi:10.1080/14660820050515197 11464953

[51] Géral C , Angelova A , Lesieur S . From molecular to nanotechnology strategies for delivery of neurotrophins: emphasis on brain‐derived neurotrophic factor (BDNF). Pharmaceutics. 2013;5:127‐167. doi:10.3390/pharmaceutics5010127 24300402 PMC3834942

[52] Yang J , Siao CJ , Nagappan G , et al. Neuronal release of proBDNF. Nat Neurosci. 2009;12:113‐115. doi:10.1038/nn.2244 19136973 PMC2737352

[53] Kallunki T , Barisic M , Jäättelä M , Liu B . How to choose the right inducible gene expression system for mammalian studies? Cells. 2019;8:796. doi:10.3390/cells8080796 PMC6721553 31366153

[54] Dogbevia GK , Roβmanith M , Sprengel R , Hasan MT . Flexible, AAV‐equipped genetic modules for inducible control of gene expression in mammalian brain. Mol Ther Nucleic Acids. 2016;5:e309. doi:10.1038/mtna.2016.23 27070301 PMC5014524

