# Supplementary material for: Engineering a switchable single‐chain TEV protease to control protein maturation in living neurons
Source: Bioeng Transl Med. 2022 Feb 22;7(2):e10292. doi: 10.1002/btm2.10292 (PMC9115699; doi:10.1002/btm2.10292)
Supplement: Supplementary file 1 — Appendix S1: Supporting Information [file BTM2-7-e10292-s001.docx]

**Supporting Information**

**Supplementary Table 1**.

Mean values of protease activity of constitutively active TEV, SPELL-TEV, uniRapR-TEV LL, uniRapR-TEV ML, uniRapR-TEV SL, uniRapR-TEV NL, evaluated by monitoring the migration of the synthetic Cerulean-TEVcs-YPet protein cleavage in SDS-PAGE experiments.

**Supplementary Table 2**.

Mean values of EGFP expression in the presence of veh or 2 μM rap for 6 h evaluated by quantifying fluorescence intensity signal.

| pro-BDNF (MRV🡪ENL)_Fw | GGGTCAGAGTGGCGCCGGAGATTCTCGGACATGTTTGCAGCATCT |
| --- | --- |
| pro-BDNF (MRV🡪ENL)_Rv | AGATGCTGCAAACATGTCCGAGAATCTCCGGCGCCACTCTGACCC |
| pro-BDNF (RRH🡪YFQ)_Fw | CCCTCGGCGGGCAGGGTCAGACTGGAAATAGAGATTCTCGGACATGTTTGC |
| pro-BDNF (RRH🡪YFQ)_Rw | GCAAACATGTCCGAGAATCTCTATTTCCAGTCTGACCCTGCCCGCCGAGGG |
| Ins_ uniRapR-TEV _Fw | CAACTTCCAAACTAAGAGCACCTGCGTGG |
| Ins_ uniRapR-TEV _Rv | TGACACCATGCTAGACATTTCCAGTTTTAGAAGCT |
| Open_TEV120/121_Fw | ATGTCTAGCATGGTGTCAGACACTAG |
| Open_TEV120/121_Rv | GCTCTTAGTTTGGAAGTTGGTTGT |
| Ins_ uniRapR-secTEV _Fw | CCAACTTCCAGACCAAATCCACCTGCGTGGTGCACTACACCGG |
| Ins_ uniRapR-secTEV _Rv | TGACACCATGCTAGACATTTCCAGTTTTAGAAGCT |
| Open_secTEV120/121_Fw | ATGAGCTCCATGGTCTCCGACAC |
| Open_secTEV120/121_Rv | GGATTTGGTCTGGAAGTTGGTAG |

**Supplementary Table 3**. Sequences of the primers used to obtain uniRapR-TEVs and pro-TEVcs-BDNF constructs. Ins: primers used for amplifying insertion uniRapR; Open: primers used for amplifying and open TEV vectors.

| Antibodies | Source | Identifier |
| --- | --- | --- |
| Mouse monoclonal anti-Flag-M2 antibody | Sigma-Aldrich | Cat#. F1804 |
| Flag-M2 agarose | Sigma-Aldrich | Cat#. A2220 |
| Mouse monoclonal anti- ERK1/2 | Santa Cruz Biotechnology | Cat#. sc-514302 |
| Rabbit Monoclonal anti-Phospho-ERK1/2 | Sigma-Aldrich | Cat#. AW39R |
| Mouse monoclonal anti-SV5 | Thermo Fisher Scientific | Cat#. **MA1-34099** |
| Rabbit Polyclonal anti-GFP | Thermo Fisher Scientific | Cat#. A-11122 |
| Mouse monoclonal anti-GAPDH | Cell Signaling | Cat#. 97166 |

**Supplementary Table 4**. Antibodies

**
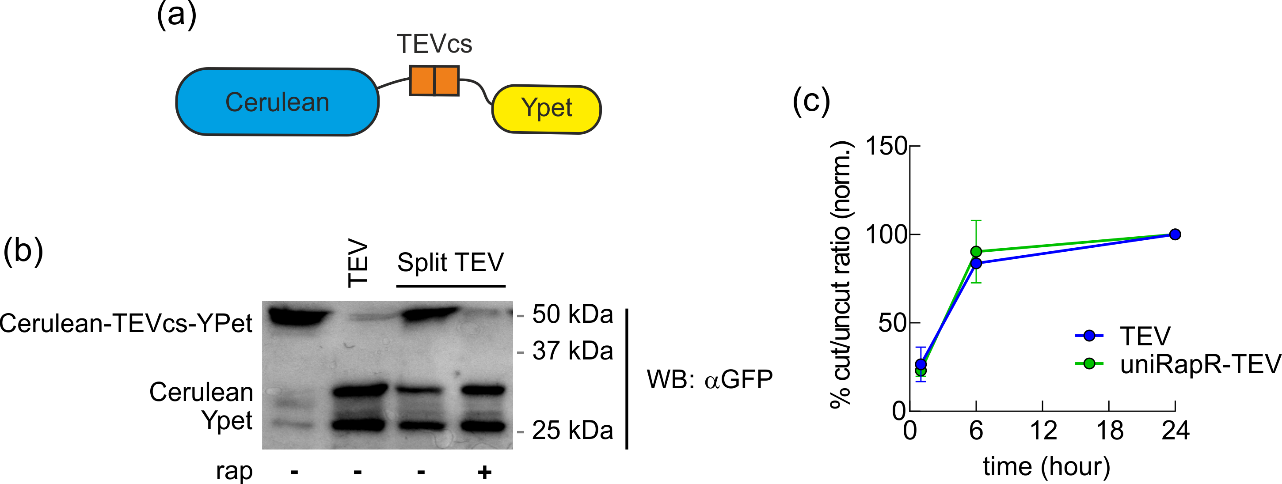
**

**Supplementary FIGURE 1**

**Background activity of split TEV construct and kinetics of protease activity of constitutively active TEV and uniRapR-TEV.** (a) Schematic diagram showing the synthetic Cerulean-TEVcs-YPet construct used to detect cytosolic TEV activity in living cells. HEK293T cells transfected with Cerulean-TEVcs-YPet and either constitutively active TEV or Split TEV were treated with EtOH or 2 μM rap for 6 h. (b) The cleaved products were probed on a western blot with GFP antibody. Split TEV showed substantial background activity, which was attributed to an inherent affinity between TEV fragments. (c) Normalized mean values of TEV catalytic activity evaluated in *in vitro* assay using purified TEV, uniRapR-TEV and Cerulean-TEVcs-Ypet as substrate. The *in vitro* proteolytic activity assay showed that the substrate was cut in a similar time-dependent manner with both rapamycin-activated uniRapR-TEV and TEV (% of cut/uncut ratio at 1 h normalized to 24h data: 26.58±11.95% *vs* 23.10±4.07% obtained with TEV and rapamycin-activated uniRapR-TEV, respectively; % of cut/uncut ratio at 6h normalized to 24h data: 83.65±3.42% *vs* 90.31±21.64% obtained with TEV and rapamycin-activated uniRapR-TEV, respectively).

proBDNF Mouse: X55573

proBDNF Rat: M61175

proBDNF Human: M61176

mouse MTILFLTMVISYFGCMKAAPMKEVNVHGQGNLAYPGVRTHGTLESVNGPRAGSRGLTTTS

rat MTILFLTMVISYFGCMKAAPMKEANVHGQGNLAYPAVRTHGTLESVNGPRAGSRGLTTTS

human MTILFLTMVISYFGCMKAAPMKEANIRGQGGLAYPGVRTHGTLESVNGPKAGSRGLTS--

***********************.*::***.****.*************:*******:

mouse LADTFEHVIEELLDEDQKVRPNEENHKDADLYTSRVMLSSQVPLEPPLLFLLEEYKNYLD

rat LADTFEHVIEELLDEDQKVRPNEENHKDADLYTSRVMLSSQVPLEPPLLFLLEEYKNYLD

human LADTFEHVIEELLDEDQKVRPNEENNKDADLYTSRVMLSSQVPLEPPLLFLLEEYKNYLD

*************************:**********************************

mouse AANMSMRVRRHSDPARRGELSVCDSISEWVTAADKKTAVDMSGGTVTVLEKVPVSKGQLK

rat AANMSMRVRRHSDPARRGELSVCDSISEWVTAADKKTAVDMSGGTVTVLEKVPVSKGQLK

human AANMSMRVRRHSDPARRGELSVCDSISEWVTAADKKTAVDMSGGTVTVLEKVPVSKGQLK

************************************************************

mouse QYFYETKCNPMGYTKEGCRGIDKRHWNSQCRTTQSYVRALTMDSKKRIGWRFIRIDTSCV

rat QYFYETKCNPMGYTKEGCRGIDKRHWNSQCRTTQSYVRALTMDSKKRIGWRFIRIDTSCV

human QYFYETKCNPMGYTKEGCRGIDKRHWNSQCRTTQSYVRALTMDSKKRIGWRFIRIDTSCV

************************************************************

mouse CTLTIKRGR

rat CTLTIKRGR

human CTLTIKRGR

*********

**Supplementary FIGURE 2**

**Sequence alignment of proBDNF protein sequences from mouse, rat, and human.** Amino acid sequence of proBDNF in mouse (GenBank: X55573.1), rat (GenBank: M61175.1) and human (GenBank: M61176.1). The endogenous cleavage sequence in proBDNF is shown in red.


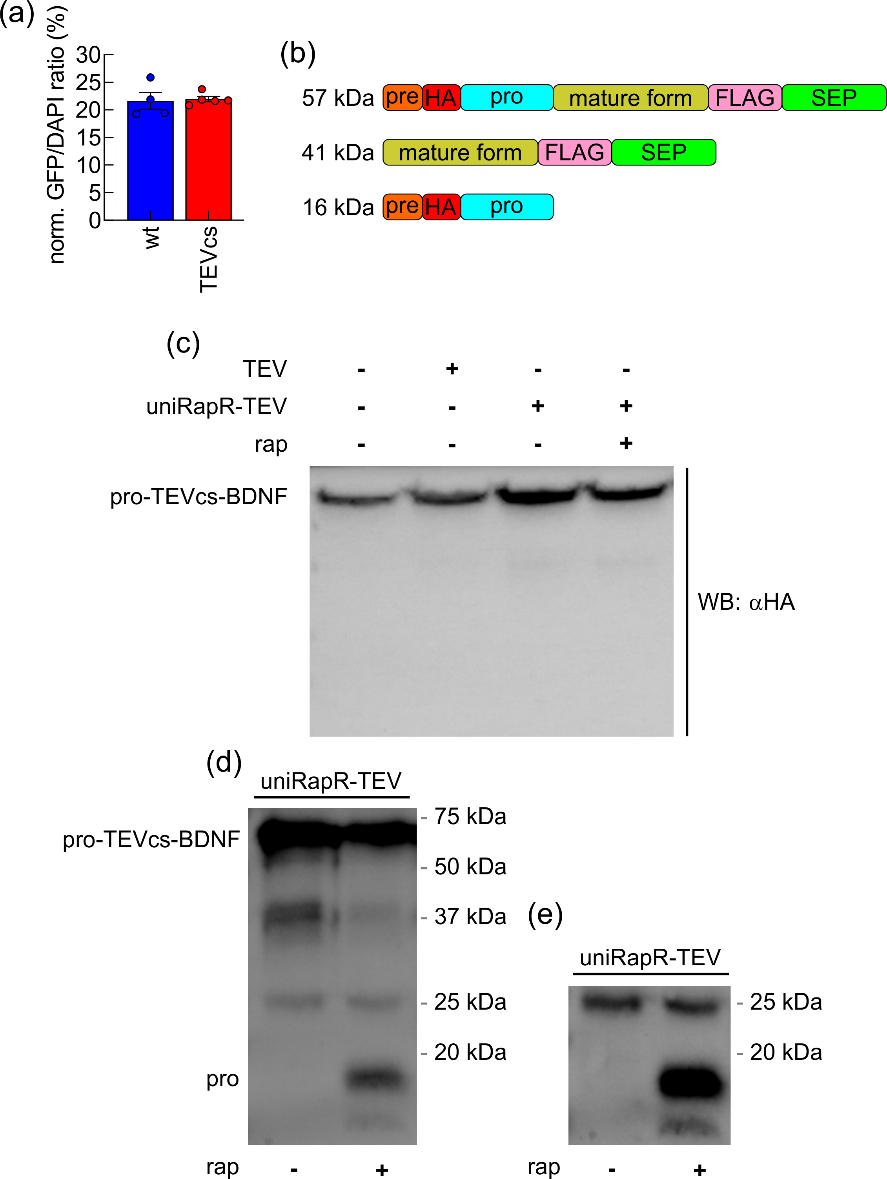


**Supplementary FIGURE 3**

**Cytosolic uniRapR-TEV is functionally inactive in the secretory pathway of mammalian cells.** (a) Quantification of immunostaining of HEK293T cells expressing the wt proBDNF and mutant pro-TEVcs-BDNF. Mutations introduced into proBDNF (from MRVRRH to ENLYFQ) did not alter the % of GFP-positive cells. (b) Schematic representation of the Flag- and SEP-tagged proBDNF and fragments resulting from protease activity. (c) Representative western blot of lysates of HEK293T cells transfected with pro-TEVcs-BDNF and the cytosolic versions of TEV or uniRapR-TEV in absence or presence of rapamycin (rap). Pro-TEVcs-BDNF was cut by neither constitutively active cytosolic TEV nor uniRapR-TEV in living cells. (d) UniRapR-TEV cleavage of pro-TEVcs-BDNF *in vitro*. Purified pro-TEVcs-BDNF was incubated with purified uniRapR-TEV in the presence of EtOH or rap. The cleaved products were probed on a western blot with an antibody to HA. UniRapR-TEV plus rap, but not uniRapR-TEV with EtOH, cut pro-TEVcs-BDNF producing pro-peptide and BDNF. (e) The same membrane was subjected to a longer exposure time.

1. **AMINO ACID SEQUENCES**

**TEV**

GESLFKGPRDYNPISSTICHLT**N**ESDGHTTSLYGIGFGPFIITNKHLFRRNNGTLLVQSLHGVFKVKNTTTLQQHLIDGRDMIIIRMPKDFPPFPQKLKFREPQREERICLVTTNFQTKSRMSSMVSDTSCTFPSSDGTFWKHWIQTKDGQCGNPLVSTRDGFIVGIHSASNF**T**NTNNYFASVPKNFMELLTNQEAQQWVSGWRLNADSVLWGGHKVFMVKPEEPFQPVKEATQLMN*

**secTEV**

MGWSLILLFLVAVATGVHSQGAQGESLFKGPRDYNPISSTICHLT**Q**ESDGHTTSLYGIGFGPFIITNKHLFRRNNGTLLVQSLHGVFKVKNTTTLQQHLIDGRDMIIIRMPKDFPPFPQKLKFREPQREERICLVTTNFQTKSMSSMVSDTS**S**TFPSSDGTFWKHWIQTKDGQCGNPLVSTRDGFIVGIHSASNF**G**NTNNYFASVPKNFMELLTNQEAQQWVSGWRLNADSVLWGGHKVFMSKPEEPFQPVKEATQLMNEGGLE*

**uniRapR-TEV -** Long Linker (LL): GGSGGG; Medium Linker (ML): GGS; Small Linker (SL): G

GESLFKGPRDYNPISSTICHLT**N**ESDGHTTSLYGIGFGPFIITNKHLFRRNNGTLLVQSLHGVFKVKNTTTLQQHLIDGRDMIIIRMPKDFPPFPQKLKFREPQREERICLVTTNFQTKS(Linker)TCVVHYTGMLEDGKKFDSSRDRNKPFKFMLGKQEVIRGWEEGVAQMSVGQRAKLTISPDYAYGATGHGSGSGSGVKDLLQAWDLYYHVFRRISGPPGPGSGLWHEMWHEGLEEASRLYFGERNVKGMFEVLEPLHAMMERGPQTLKETSFNQAYGRDLMEAQEWCRKYMKSGSSGGSGSGIIPPHATLVFDVELLKLE(Linker)RMSSMVSDTSCTFPSSDGTFWKHWIQTKDGQCGNPLVSTRDGFIVGIHSASNF**T**NTNNYFASVPKNFMELLTNQEAQQWVSGWRLNADSVLWGGHKVFMVKPEEPFQPVKEATQLMN*

**uniRapR-secTEV -** Long Linker (LL): GGSGGG; Medium Linker (ML): GGS; Small Linker (SL): G MGWSLILLFLVAVATGVHSQGAQGESLFKGPRDYNPISSTICHLT**Q**ESDGHTTSLYGIGFGPFIITNKHLFRRNNGTLLVQSLHGVFKVKNTTTLQQHLIDGRDMIIIRMPKDFPPFPQKLKFREPQREERICLVTTNFQTK(Linker)TCVVHYTGMLEDGKKFDSSRDRNKPFKFMLGKQEVIRGWEEGVAQMSVGQRAKLTISPDYAYGATGHGSGSGSGVKDLLQAWDLYYHVFRRISGPPGPGSGLWHEMWHEGLEEASRLYFGERNVKGMFEVLEPLHAMMERGPQTLKETSFNQAYGRDLMEAQEWCRKYMKSGSSGGSGSGIIPPHATLVFDVELLKLE(Linker)MSSMVSDTS**S**TFPSSDGTFWKHWIQTKDGQCGNPLVSTRDGFIVGIHSASNF**G**NTNNYFASVPKNFMELLTNQEAQQWVSGWRLNADSVLWGGHKVFMSKPEEPFQPVKEATQLMNEGGLE*

1. **NUCLEOTIDE SEQUENCES**

**TEV**

ggagaaagcttgtttaaggggccgcgtgattacaacccgatatcgagcaccatttgtcatttgacg**aat**gaatctgatgggcacacaacatcgttgtatggtattggatttggtcccttcatcattacaaacaagcacttgtttagaagaaataatggaacactgttggtccaatcactacatggtgtattcaaggtcaagaacaccacgactttgcaacaacacctcattgatgggagggacatgataattattcgcatgcctaaggatttcccaccatttcctcaaaagctgaaatttagagagccacaaagggaagagcgcatatgtcttgtgacaaccaacttccaaactaagagcatgtctagcatggtgtcagacactagt**ttg**cacattcccttcatctgatggtatattctggaagcattggattcaaaccaaggatgggcagtgtggcagtccattagtatcaactagagatgggttcattgttggtatacactcagcatcgaatttc**acc**aacacaaacaattatttcacaagcgtgccgaaaaacttcatggaattgttgacaaatcaggaggcgcagcagtgggttagtggttggcgattaaacgctgactcagtattgtgggggggccataaagttttcatggtgaaacctgaagaaccttttcagccagttaaggaagcgactcaactcatgaattga

**secTEV**

atgggctggagcctgatcctcctgttcctcgtcgctgtggctacaggtgtgcactctcagggcgcgcaaggggaaagcctgttcaagggaccaagggactacaatccaatctcctcaactatctgccacctgact**cag**gaaagcgacggacataccacatctctgtacggaattggcttcgggcccttcatcattactaacaagcacctgtttcggagaaacaatggcaccctgctggtgcagagtctgcacggggtgttcaaggtcaaaaatactaccacactgcagcagcatctgattgacggacgagatatgatcattatccggatgccaaaggacttccccccttttccccagaagctgaagttccgggagccccagagggaggaacgcatctgcctggtgactaccaacttccagaccaaatccatgagctccatggtctccgacacctct**tct**acattcccttctagtgatggcatcttctggaagcactggatccagacaaaagacggacagtgcggcagtccactggtgtcaaccagagatgggtttattgtcggaatccattcagccagcaacttc**gga**aatactaacaattacttcacctctgtgcccaaaaacttcatggagctgctgactaatcaggaagcacagcagtgggtgagcggatggcgcctgaatgctgattccgtgctgtggggcgggcataaggtcttcatgagcaaacctgaagagccatttcagcccgtcaaggaagccacccagctgatgaacgaagggggcctggaaggttaa

**uniRapR-TEV-** Long Linker (LL): ggtggatcagggggaggt; Medium Linker (ML): ggtggatca; Small Linker (SL): ggt

ggagaaagcttgtttaaggggccgcgtgattacaacccgatatcgagcaccatttgtcatttgacg**aat**gaatctgatgggcacacaacatcgttgtatggtattggatttggtcccttcatcattacaaacaagcacttgtttagaagaaataatggaacactgttggtccaatcactacatggtgtattcaaggtcaagaacaccacgactttgcaacaacacctcattgatgggagggacatgataattattcgcatgcctaaggatttcccaccatttcctcaaaagctgaaatttagagagccacaaagggaagagcgcatatgtcttgtgacaaccaacttccaaactaagagc(Linker)acctgcgtggtgcactacaccgggatgcttgaagatggaaagaaatttgattcctcccgggacagaaacaagccctttaagtttatgctaggcaagcaggaggtgatccgaggctgggaagaaggggttgcccagatgagtgtgggtcagagagccaaactgactatatctccagattatgcctatggtgccactgggcacggttcgggctccggatcaggcgtcaaggacctcctccaagcctgggacctctattatcatgtgttccgacgaatctcaggtcctccaggacctggatcaggtctctggcatgagatgtggcatgaaggcctggaagaggcatctcgtttgtactttggggaaaggaacgtgaaaggcatgtttgaggtgctggagcccttgcatgctatgatggaacggggcccccagactctgaaggaaacatcctttaatcaggcctatggtcgagatttaatggaggcccaagagtggtgcaggaagtacatgaaatcagggtcatcagggggctccggatcaggcatcatcccaccacatgccactctcgtcttcgatgtggagcttctaaaactggaa(Linker)atgtctagcatggtgtcagacactagtttgcacattcccttcatctgatggtatattctggaagcattggattcaaaccaaggatgggcagtgtggcagtccattagtatcaactagagatgggttcattgttggtatacactcagcatcgaatttc**acc**aacacaaacaattatttcacaagcgtgccgaaaaacttcatggaattgttgacaaatcaggaggcgcagcagtgggttagtggttggcgattaaacgctgactcagtattgtgggggggccataaagttttcatggtgaaacctgaagaaccttttcagccagttaaggaagcgactcaactcatgaattaa

**uniRapR-secTEV-** Long Linker (LL): ggtggatcagggggaggt; Medium Linker (ML): ggtggatca; Small Linker (SL): ggt

atgggctggagcctgatcctcctgttcctcgtcgctgtggctacaggtgtgcactctcagggcgcgcaaggggaaagcctgttcaagggaccaagggactacaatccaatctcctcaactatctgccacctgact**cag**gaaagcgacggacataccacatctctgtacggaattggcttcgggcccttcatcattactaacaagcacctgtttcggagaaacaatggcaccctgctggtgcagagtctgcacggggtgttcaaggtcaaaaatactaccacactgcagcagcatctgattgacggacgagatatgatcattatccggatgccaaaggacttccccccttttccccagaagctgaagttccgggagccccagagggaggaacgcatctgcctggtgactaccaacttccagaccaaatcc(Linker)acctgcgtggtgcactacaccgggatgcttgaagatggaaagaaatttgattcctcccgggacagaaacaagccctttaagtttatgctaggcaagcaggaggtgatccgaggctgggaagaaggggttgcccagatgagtgtgggtcagagagccaaactgactatatctccagattatgcctatggtgccactgggcacggttcgggctccggatcaggcgtcaaggacctcctccaagcctgggacctctattatcatgtgttccgacgaatctcaggtcctccaggacctggatcaggtctctggcatgagatgtggcatgaaggcctggaagaggcatctcgtttgtactttggggaaaggaacgtgaaaggcatgtttgaggtgctggagcccttgcatgctatgatggaacggggcccccagactctgaaggaaacatcctttaatcaggcctatggtcgagatttaatggaggcccaagagtggtgcaggaagtacatgaaatcagggtcatcagggggctccggatcaggcatcatcccaccacatgccactctcgtcttcgatgtggagcttctaaaactggaa(Linker)atgagctccatggtctccgacacctct**tct**acattcccttctagtgatggcatcttctggaagcactggatccagacaaaagacggacagtgcggcagtccactggtgtcaaccagagatgggtttattgtcggaatccattcagccagcaacttc**gga**aatactaacaattacttcacctctgtgcccaaaaacttcatggagctgctgactaatcaggaagcacagcagtgggtgagcggatggcgcctgaatgctgattccgtgctgtggggcgggcataaggtcttcatgagcaaacctgaagagccatttcagcccgtcaaggaagccacccagctgatgaacgaagggggcctggaaggttaa

**Supplementary FIGURE 4**

**Protein and nucleotide sequences of engineered single-chain TEV variants activatable in the cytosol and in the secretory pathway.** (a) Amino acid sequence of TEV; secTEV, uniRapR-TEV and uniRapR-secTEV. N23Q-C130S-T173G mutations are in bold underlined. The secretory signal is shown in red. Spacer sequence in cerulean. UniRapR is shown in yellow and the N- and C-terminus are shown in grey and green, respectively. Linkers are in blu. S219V mutation and NEGGLE peptide represented in magenta avoid autoproteolysis in TEV variants activatable in the cytosol and in the secretory pathway, respectively. (b) Nucleotide sequences of the proteins described in (a).


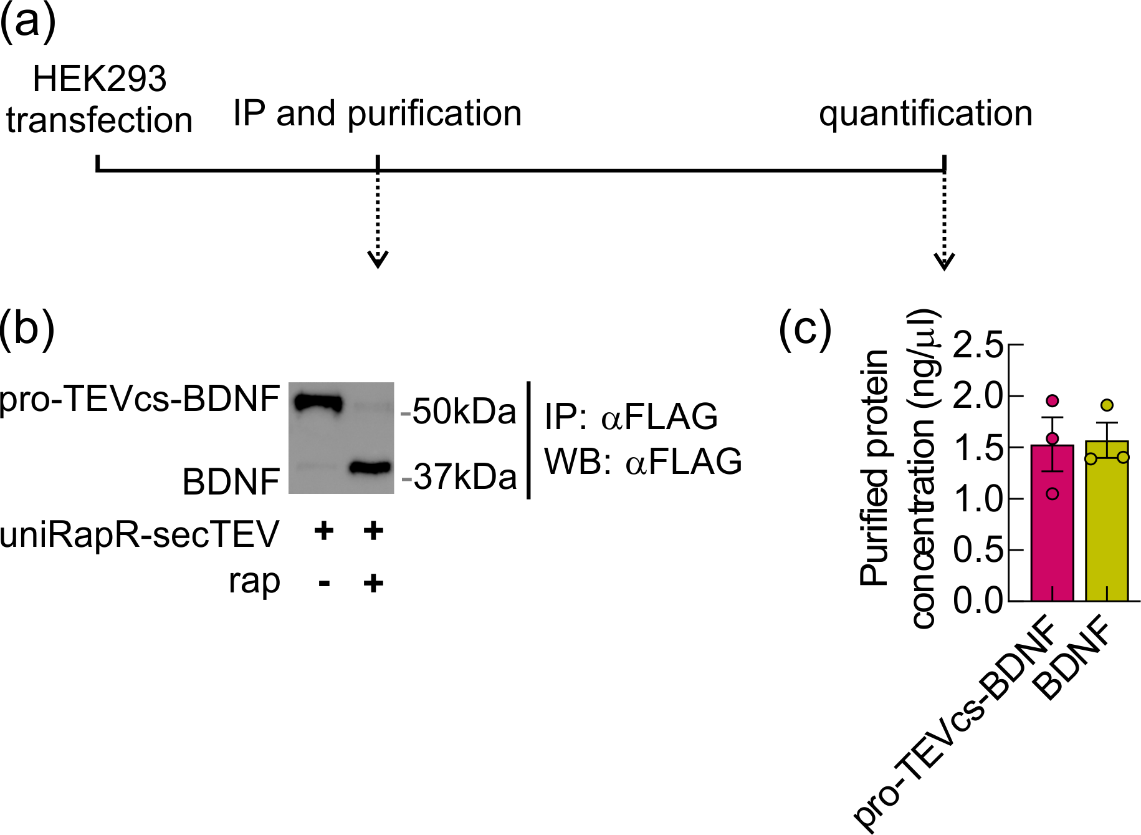


**Supplementary FIGURE 5**

**Purification and quantification of BDNF.** (a) Schematic representation of the experimental design to purify and quantify proteins from HEK293T cells transfected with pro-TEVcs-BDNF and uniRapR-secTEV. Twenty-four h after transfection, HEK293T cells were treated with 4 μM rapamycin (rap) for 6 h. Cell lysates were immunoprecipitated with Flag antibody in the presence of vehicle (-) or rap (+). After washing, proteins were purified and stored. (b) Samples were also eluted with SDS-PAGE sample buffer and immunoblotted with Flag antibody. (c) Bar graph showing mean concentrations of pro-TEVcs-BDNF (1.53±0.26 ng/μl, n=3) and BDNF (1.57±0.17 ng/μl, n=3) immunoprecipitated and purified from 3×10^6^ cells for each condition and quantified using BDNF ELISA kit. Error bars indicate SEM.


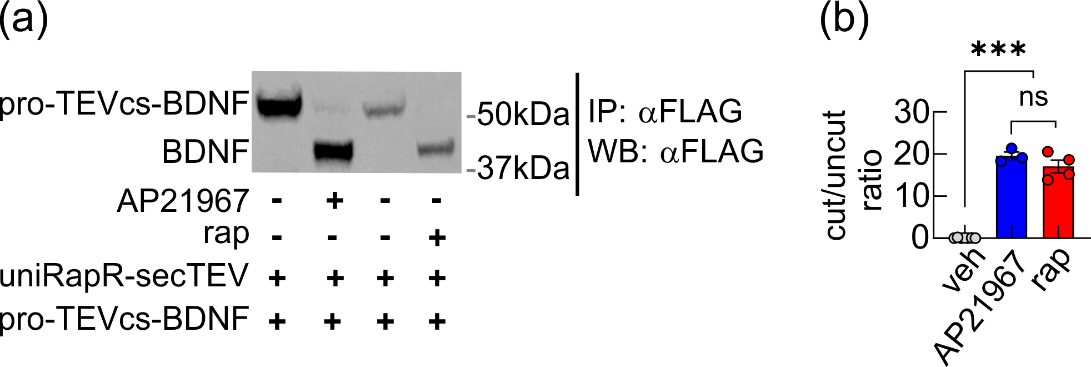


**Supplementary FIGURE 6**

**Activation of engineered single-chain TEV protease by a non-immunosuppressive analog of rapamycin.** (a) Protease activity of uniRapR-secTEV evaluated by monitoring the pro-TEVcs-BDNF protein cleavage in SDS-PAGE experiments with or without the non-immunosuppressive rapamycin (rap) analog AP21967 or rap. (b) Summary graph of the ratio between densitometric values of the upper/lower bands representing the results of the pro-TEVcs-BDNF cleavage by uniRapR-secTEV with or without AP21967 or rap (vehicle: 0.080±0.03, n=7; AP21967: 19.59 0.91, n=3; rap: 17.06 1.53, n=4). ***p < 0.0005 compared to veh condition; one-way ANOVA with the Dunnett’s *post hoc* test comparisons.

**
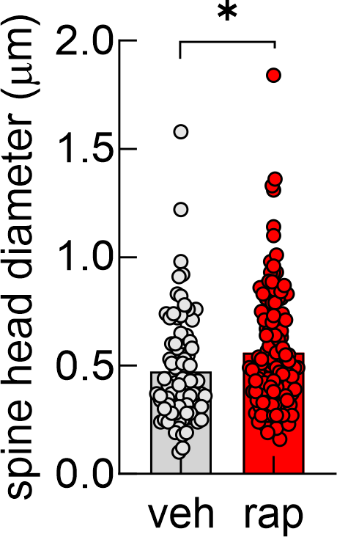
**

**Supplementary FIGURE 7**

**Mature BDNF cleaved from pro-TEVcs-BDNF promotes dendritic spines enlargement in living cells.** Bar graph showing the normalized diameter of spine head (0.47±0.02, n=91 *vs* 0.56±0.02, n=135 in neurons transfected with uniRapR-secTEV, pro-TEVcs-BDNF and dsRed2, treated with veh and rap, respectively). Analysis was performed by using Nikon Ti-E software on a total of at least 15 frames/group. Error bars indicate SEM. *p < 0.05 by Student's t test.


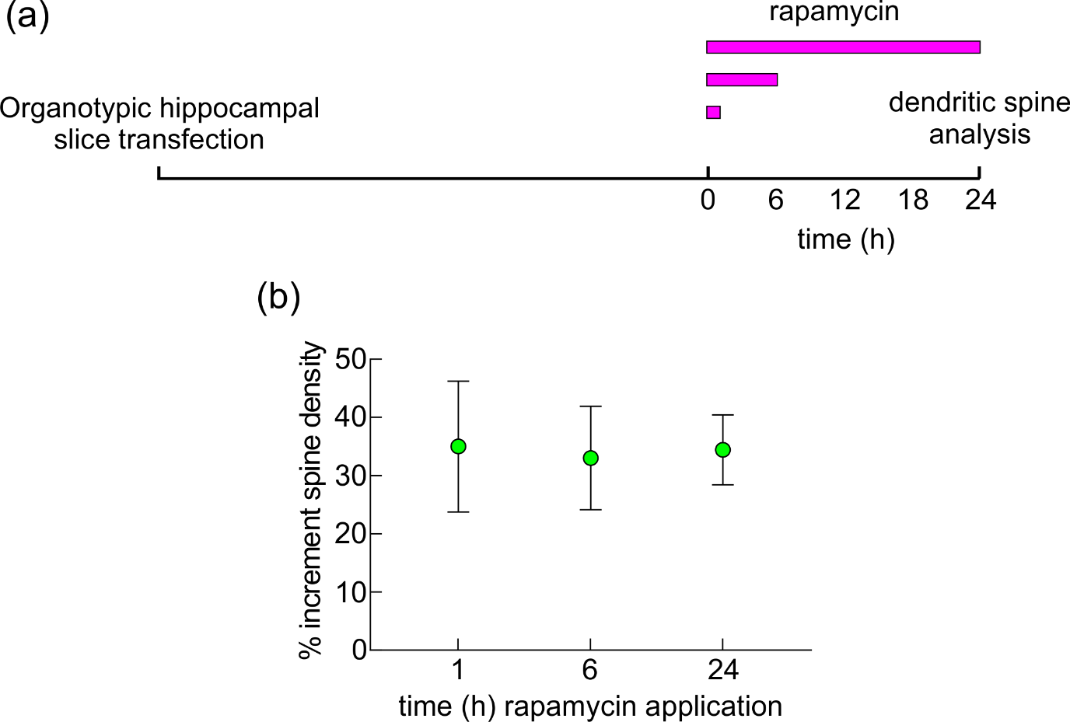


**Supplementary FIGURE 8**

**Brief uniRapR-secTEV activation was sufficient to generate a significant increase in dendritic spine density after 24 h of the beginning of the treatment.** (a) Schematic representation of the experimental design to evaluate the BDNF turnover and the rate of the shutdown of TEV activity. (b) Neurons transfected with uniRapR-secTEV and pro-TEVcs-BDNF were treated with 1 μM rapamycin for 1, 6 and 24 h. Spine density increase (as %) in rapamycin-treated neurons *vs* vehicle-treated samples was evaluated at 24 h of the beginning of the treatment (1 h: +35.27±11.23, n=16; 6 h: +32.98±8.88, n=12; 24h: +34.42±6.00, n=35). In each experiment, at least three segments (20 μm) from secondary dendrites from two neurons were analysed.
